# Specific myeloid signatures in peripheral blood differentiate active and rare clinical phenotypes of multiple sclerosis

**DOI:** 10.3389/fimmu.2023.1071623

**Published:** 2023-01-25

**Authors:** Aigli G. Vakrakou, Nikolaos Paschalidis, Eleftherios Pavlos, Christina Giannouli, Dimitris Karathanasis, Xristina Tsipota, Georgios Velonakis, Christine Stadelmann-Nessler, Maria-Eleftheria Evangelopoulos, Leonidas Stefanis, Constantinos Kilidireas

**Affiliations:** ^1^ Demyelinating Diseases Unit, 1st Department of Neurology, School of Medicine, Aeginition Hospital, National and Kapodistrian University of Athens, Athens, Greece; ^2^ Department of Neuropathology, University of Göttingen Medical Center, Göttingen, Germany; ^3^ Mass Cytometry-CyTOF Laboratory, Center for Clinical Research, Experimental Surgery and Translational Research, Biomedical Research Foundation of the Academy of Athens, Athens, Greece; ^4^ Center for Clinical Research, Experimental Surgery and Translational Research Biomedical Research Foundation of the Academy of Athens, Athens, Greece; ^5^ Division of Basic Sciences, University of Crete Medical School, Heraklion, Greece; ^6^ Research Unit of Radiology, 2nd Department of Radiology, Medical School, National and Kapodistrian University of Athens, Athens, Greece; ^7^ Department of Neurology, Henry Dunant Hospital Center, Athens, Greece

**Keywords:** multiple sclerosis, tumefactive multiple sclerosis, mass cytometry, myeloid-signature, macrophages, T cells, B cells, NK cells

## Abstract

Current understanding of Multiple Sclerosis (MS) pathophysiology implicates perturbations in adaptive cellular immune responses, predominantly T cells, in Relapsing-Remitting forms (RRMS). Nevertheless, from a clinical perspective MS is a heterogeneous disease reflecting the heterogeneity of involved biological systems. This complexity requires advanced analysis tools at the single-cell level to discover biomarkers for better patient-group stratification. We designed a novel 44-parameter mass cytometry panel to interrogate predominantly the role of effector and regulatory subpopulations of peripheral blood myeloid subsets along with B and T-cells (excluding granulocytes) in MS, assessing three different patient cohorts: RRMS, PPMS (Primary Progressive) and Tumefactive MS patients (TMS) (n=10, 8, 14 respectively). We further subgrouped our cohort into inactive or active disease stages to capture the early underlying events in disease pathophysiology. Peripheral blood analysis showed that TMS cases belonged to the spectrum of RRMS, whereas PPMS cases displayed different features. In particular, TMS patients during a relapse stage were characterized by a specific subset of CD11c+CD14+ CD33+, CD192+, CD172+-myeloid cells with an alternative phenotype of monocyte-derived macrophages (high arginase-1, CD38, HLA-DR-low and endogenous TNF-a production). Moreover, TMS patients in relapse displayed a selective CD4 T-cell lymphopenia of cells with a Th2-like polarised phenotype. PPMS patients did not display substantial differences from healthy controls, apart from a trend toward higher expansion of NK cell subsets. Importantly, we found that myeloid cell populations are reshaped under effective disease-modifying therapy predominantly with glatiramer acetate and to a lesser extent with anti-CD20, suggesting that the identified cell signature represents a specific therapeutic target in TMS. The expanded myeloid signature in TMS patients was also confirmed by flow cytometry. Serum neurofilament light-chain levels confirmed the correlation of this myeloid cell signature with indices of axonal injury. More in-depth analysis of myeloid subsets revealed an increase of a subset of highly cytolytic and terminally differentiated NK cells in PPMS patients with leptomeningeal enhancement (active-PPMS), compared to those without (inactive-PPMS). We have identified previously uncharacterized subsets of circulating myeloid cells and shown them to correlate with distinct disease forms of MS as well as with specific disease states (relapse/remission).

## 1 Introduction

The inflammatory response leading to demyelination in multiple sclerosis (MS) is a result of multi-directional feedback involving central nervous system (CNS)-resident cells and infiltrating immune cells (1). Findings from animal models and immunological studies in patients with MS indicate that peripheral immune responses targeting the CNS drive disease during the early phases, whereas immune reactions within the CNS dominate the progressive phases (2–5). Chronic inflammation, which occurs behind a closed blood–brain barrier with activation of microglia and continued involvement of T and B cells, is a hallmark pathophysiological feature, especially in patients with Primary Progressive Multiple Sclerosis (PPMS).

A major contribution to MS pathogenesis has been attributed to perturbations of the adaptive immune system with the roles of antigen-specific encephalitogenic T cells and clonally expanded B cells extensively addressed (6–12). The role of innate immunity has been predominantly assessed in the inflamed brain tissue with studies describing the activation status of infiltrating macrophages/microglia within the CNS of patients and in animal models of MS, showing that infiltrating blood monocytes exert a crucial role in the effector phase of the disease. In humans, genome-wide association studies (GWAS) unraveled causal variants for MS in genes expressed in multiple cell types, including monocytes/macrophages and microglial cells (13).

In MS, peripheral blood monocyte abnormalities have been described in studies reporting variable and sometimes contradictory results. One important factor accounting for that could be the timing of immune phenotyping relative to disease activity. Increased percentage of circulating CD16+ (non-classical and intermediate) monocytes has been reported in some studies, whereas others point to a more compartmentalized distribution, with a lower percentage of circulating CD16+ cells and an increased percentage in the cerebrospinal fluid (CSF) of patients (14–16). Circulating monocytes from patients with MS secrete more pro-inflammatory (e.g., IL-6 and IL-12) cytokines and express more co-stimulatory molecules, a phenotype favoring proinflammatory T-cell responses (17, 18).

Flow cytometry used in previous studies permits the simultaneous detection of a limited number of fluorophores tagged with antibodies. As such, the need for multiparametric analysis at a single-cell level has been overcome with new technologies like multiplexed single-cell mass cytometry (cytometry by time of flight, CyTOF). Subgrouping of MS patients in clinical disease forms and activity are critical factors for the design of studies in an attempt to limit disease heterogeneity. Recent studies that applied CyTOF technology for the analysis of peripheral blood mainly focusing on early MS, have shown phenotypic changes mainly in T cells, an increased abundance of T-bet-expressing B cells and a CD206+ classical monocyte subset, as well as a specific B cell subset only in the CSF of such patients (19, 20).

There are still controversies regarding cell type heterogeneity in MS pathogenesis (19, 21–24). An unmet need in the field of MS research is the identification of biomarkers and cell type signatures able to differentiate among different and rare disease subgroups. This knowledge could permit the design of better-targeted therapies. Towards this direction, there needs to be more evidence regarding biomarkers in the peripheral blood of patients with PPMS that could reflect *in vivo* active disease pathology of the CNS. PPMS is the less prevalent disease form of MS and the most difficult to treat efficiently, with a more insidious clinical disease course without relapses and less radiological activity (e.g. gadolinium-enhancing lesions). In the present study, we stratified our PPMS patients into disease subgroups based on the extent of the leptomeningeal enhancement, as determined by MRI criteria, to capture radiological aspects of disease activity that could reflect disease-relevant neuropathology disease features (1).

Moreover, patients with tumefactive demyelinating lesions (TDL) have never been grouped for differential phenotypic analysis. TDL can emerge as part of MS pathophysiology (Tumefactive MS - TMS) during the disease course or as the initial presenting radiographic feature. TMS is considered one of the rare MS variants that have not yet been studied in depth, due to the lack of large patient cohorts. Also, there are no clear clinical/serological and/or radiographical biomarkers assessing the risk for disease evolution and conversion to clinically definite MS. This information is critically important because it determines our further therapeutic strategies after the first TDL appearance (25, 26). Immunopathology studies in the past have not found substantial differences with classical MS, except for a more intense macrophage inflammatory response with prominent phagocytosis of myelin and more prominent dystrophic changes in astrocytes. Nevertheless, whether a soluble marker or a previously unidentified cell subset could account for the greater CNS inflammatory component observed in TMS is not known.

In-depth characterization of cell types in rare forms of MS like PPMS and TMS under active and inactive disease stages along with differential analysis with classical MS will help us to biologically stratify these clinical disease forms and identify potential qualitative and/or quantitative differences from classical disease forms. For this reason, we performed an in-depth analysis of peripheral blood mononuclear cells (PBMCs) isolated from healthy controls and patients with different forms of MS using multiplexed single-cell mass cytometry combined with automated algorithm-based computational analysis tools.

## 2 Material and methods

### 2.1 Patient cohort

This study was registered and approved by the Ethics Committee of Aeginition Hospital (number 7143/08.09.2021). All participants provided written informed consent before participation in the study. Blood samples were obtained from 32 patients with MS and 10 age- and sex-paired healthy controls (HD). The demographic and clinical data of the patients with early MS and HD included in this study are summarized in **Table 1**. Inclusion criteria were: i) age > 18 years old, ii) MS diagnosis according to the 2017 McDonald criteria at the last visit, iii) sampled during either disease remission or during clinical neurological relapse (defined by the appearance of new clinical symptoms related to CNS pathology, for a period of 24 hours or more) and iv) with at least one visit/year during the follow-up, v) patients with one or more large demyelinating plaques (TDL) on brain magnetic resonance imaging (MRI), either as the first clinical event or during the follow-up period of their MS course (named TMS patients). Brain biopsy was available in 4 patients. We excluded patients with Balo-like lesions, as well as pediatric MS patients, and patients with acute demyelinating encephalomyelitis and neuromyelitis optica (NMO). Requisites for TDLs were lesion size ≧̸2 cm in diameter, with or without perilesional edema, mass effect, and/or contrast enhancement. Cerebrospinal fluid (CSF) test included white blood cell (WBC) count, total protein level, glucose level, IgG index (the normal IgG index reference was <0.65), and oligoclonal band (OCB) evaluation. All patients included in the study were negative for anti-myelin oligodendrocyte glycoprotein (MOG), anti-Aquaporin 4 (AQ4), and antinuclear (ANA) antibodies. Other exclusion criteria were the presence of peripheral nervous system involvement, other autoimmune comorbidities or systemic diseases, prior radiation exposure, and radiological or clinical data indicative of an ensuing neoplastic or paraneoplastic process.

**Table 1:** Main characteristics (clinical, radiological, and laboratory) of the participants included in the study.

	Healthy controls	MS total cohort	PPMS	Non-Tumefactive/non-progressive MS	TMS	Statistical differences among groups
N (variables tested)	10	32	8	10	14	N/A
Demographic features						
Sex: female/male (male %)	7/3 (30)	18/14 (44)	4/4 (50)	5/5 (50)	9/5 (36)	N/A
Age (mean ± SD)	38 (8.21)	43 (19.08)	51 (11.19)	41.9 (30.82)	39 (11.40)	NS
Clinical features						
Disease duration (mean ± SD)	N/A	5.32 (4.86)	6.38 (3.96)	6.113 (6.97)	4.09 (3.65)	NS
Number of patients in clinical relapse (% of total in each group)	N/A	12 (37.5)	0 (0)	5 (50)	7 (50)	N/A
Time between the last relapse and sample collection (mean ± SD)	N/A	19 (14.96)	N/A	16.8 (9.18)	20.57 (18.64)	N/A
EDSS score (mean ± SD)	N/A	3.13 (1.46)	4.06 (1.32)	2.42 (1.24)	2.8 (1.42)	NS
Treatment history (the last 2 years from sample collection)						
Number of patients under treatment with DMTs (% of total in each group)	N/A	11 (34.4)	0 (0)	4 (40)	7 (50)	N/A
Acute treatment/during disease exacerbation						
IVMP (percent)	N/A	3 (9.4)	0	1 (10)	2 (14,3)	N/A
Days from IVMP (mean ± SD)	N/A	35.33 (21.46)	0	60	23	N/A
Chronic treatment (the last 2 years from sample collection)						
Glatiramer acetate (percent)	N/A	5 (15.6)	0 (0)	3 (30)	2 (14,2)	N/A
Anti-CD20 (percent)	N/A	5 (15.6)	0 (0)	1 (16.67)	4 (28.5)	N/A
Anti-CD20 (months from last infusion/mean ± SD)	N/A	3.92 (2.63)	N/A	7.07	3.13 (2.25)	N/A
Mitoxantrone (percent)	N/A	1 (3.1)	0 (0)	0 (0)	1 (7.1)	N/A
Disease course						
CIS and/or monophasic during follow-up (percent)	N/A	4 (12.5)	0 (0)	0 (0)	4 (28.5)	N/A
Progressive (percent)	N/A	8 (25)	10 (100)	0 (0)	0 (0)	N/A
Relapsing–remitting (percent)	N/A	20 (62.5)	0 (0)	10 (100)	10 (71.4)	N/A
Radiological features						
Number of patients with at least one GD-enhancing lesion	N/A	12 (37.5)	0	5 (50)	7 (50)	N/A
CSF study (where available)						
CSF mononuclear cells (x10^6/L) (mean ± SD)	N/A	5.24 (7.5)	5.33 (10.73)	4.4 (4.34)	5.33 (10.73)	N/S
Protein (mean ± SD)	N/A	50.61 (34.83)	44.32 (18.23)	40.60 (10.06)	59.39 (47.94)	N/S
IgG index (mean ± SD)	N/A	0.86 (0.35)	0.736 (0.26)	0.89 (0.33)	0.93 (0.421)	N/S
Presence of OCBs (%)	N/A	22/26 (84.6)	8/8 (100)	7/7 (100)	7/11 (63.6)	Kruskal-Wallis test: p=0.0451
Complete Blood Count (Peripheral blood)						
Variables		**N=30**	**N=8**	**N=8**	**N=14**	**Comparisons**
NEU (%) (mean ± SD)	N/A	63.97 (9.97)	59.11 (11.67)	60.68 (8.19)	68.63 (8.23)	NS
LYM (%) (mean ± SD)	N/A	27.09 (9.43)	31,53 (11.14)	30,68 (7.75)	22.5 (7.51)	NS
MONO (%) (mean ± SD)	N/A	6.81 (1.96)	6.27 (1.87)	6.6 (2.08)	7.236 (1.99)	NS
NEU (10^9/L) (mean ± SD)	N/A	4.82 (1.53)	5.08 (1.81)	4.1 (1.22)	5.09 (1.48)	NS
LYM (10^9/L) (mean ± SD)	N/A	1.96 (0.79)	2.57 (0.81)	2.02 (0.58)	1.58 (0.50)	B vs D; p=0,01
MONO (10^9/L) (mean ± SD)	N/A	0.50 (0.166)	0.51 (0.13)	0.43 (0.14)	0.53 (0.19)	NS

Age: in years at sample collection, Disease duration: in years (mean ± SD) until sample collection, Number of patients in clinical relapse: during sample collection, Time: time elapsed between the beginning of the last relapse and sample collection expressed in days (only for those in relapse at sample collection), EDSS score: at sample collection, Number of patients under treatment with DMTs: during sample collection, IVMP: at least one cycle of IVMP during the last 2 months from sample collection, Days from IVMP: days elapsed from last IVMP cycle to sample collection (mean ± SD), Number of patients with at least one GD-enhancing lesion; refers to the MRI closer to sample collection.

CIS, clinically isolated syndrome; CSF, cerebrospinal fluid; DMT, disease-modifying treatment (including steroids) within the last 6 months; EDSS, Expanded Disability Status Scale; IVMP, intravenous pulse methylprednisolone; MRI, magnetic resonance imaging; MS, multiple sclerosis; OCB, oligoclonal bands; PPMS, primary progressive multiple sclerosis; SD, standard deviation; TMS, Tumefactive multiple sclerosis; GD, gadolinium; Anti-CD20, Anti-CD20 monoclonal antibodies used to achieve B cell depletion; WBC, white blood cells, NEU: neutrophils; LYM, lymphocytes; MONO, monocytes; vs, versus, N/A; non-applicable; NS, not significant.

### 2.2 Mass cytometry

Antibodies were purchased either already conjugated to metals (Maxpar Direct Immunophenotyping Assay, MDIPA, 30 marker backbone in lyophilized form, Standard Biotools (SB) Inc. (formerly Fluidigm), San Francisco, CA (27) or in unconjugated forms (Biolegend). The MDIPA backbone was complemented with in-house conjugated antibodies against CD192, CD206, CD172a/b, TNFa, CD24, CD86, Arginase-1, CD40, IL-10, CD138, CD33, IL-6, CD163 and CD274/PD-L1. This was performed with either cadmium (Cd) (Maxpar MCP9 Antibody Labeling Kit) or lanthanide (Ln) metals (Maxpar X8 Antibody Labeling Kit), according to manufacturer’s instructions (both from SB), San Francisco, CA). The antibody panel used, clones and metal tags are provided in **Supplementary Table 1**. The in-house conjugated antibodies were validated and titrated according to the manufacturer’s instructions (Maxpar Antibody Labeling, PRD002, SB Inc., San Francisco, CA). An example of such validation can be found in **Supplementary Figure 1**. PBMCs were isolated by density-gradient centrifugation (Lymphoprep, STEMCELL Technologies, Inc.) and cryopreserved in FBS/DMSO 10%. For staining, PBMCs were thawed in prewarmed RPMI supplemented with 10% FBS, washed twice and then resuspended in fresh medium. For live/dead cell discrimination, cells were stained with 1 μM Cisplatin Cell-ID™ (SB), San Francisco, CA) and washed with Maxpar cell staining buffer (CSB) followed by a blocking step (Human TruStain FcX, Biolegend). Then, cells were stained for cell surface markers with the MDIPA backbone as well as CD192, CD206, CD172a/b, CD24, CD86, Arginase I, CD40, CD138, CD33, CD163 and PD-L1 according to the manufacturer’s instructions (**Supplementary Table 1**) followed by two washes with CSB, fixation for 20 min at RT with Maxpar Fix buffer, and two washes with Maxpar permeabilization buffer (Perm-S buffer). For intracellular staining, fixed and permeabilized cells were stained in 100 μl final volume with antibodies against IL-6. IL-10 and TNFa for 30 min (**Supplementary Table 1**). Finally, cells were stained in DNA intercalator solution (1:1000 dilution of 125 μM Cell-ID™ Intercalator-Ir), in Maxpar Fix and Perm buffer (all from SB), San Francisco, CA). The following day, cells were washed with CSB buffer and Cell Acquisition Solution (CAS). Immediately before the acquisition, cells were resuspended with EQ Passport beads (1:10 dilution). To maximize data quality, the acquisition rate on the Helios™ system (SB), South San Francisco, CA, USA) was maintained at a rate of 350 to 400 events/s. Acquired data were normalized using Passport beads (SB) method) with CyTOF software (version 10.7.1014). Prior to analysis, we performed data cleanup, sample quality check and batch effect control (**Supplementary Figures 2**, **3**). We used bivariate dot plots for these analyses in FlowJo™ v10.8 Software (BD Life Sciences, Franklin Lakes, NJ, USA).

### 2.3 Flow cytometry

Cryopreserved PBMC were thawed and twice washed with CSB buffer followed by a blocking step as described above. Then cells were stained for cell surface markers (CD11c, CD14, CD3, CD19, CD56), according to manufacturer instructions followed by two washes with CBS buffer. Finally, cells were suspended on PBS for acquisition on BD FACSAria™ III (for antibodies see **Supplementary Table 1**, and for gating strategy **Supplementary Figure 11A**).

### 2.4 Serum neurofilament light chain assessment

Serum neurofilament light chain (NfL) measurement was performed by Simoa (single molecule array) on a Simoa HD-X analyzer (Quanterix Corporation, 900 Middlesex Tumpike, Billerica, MA, USA).

### 2.5 Data analysis

Normalized, manually cleaned-up and gated singlet viable events from all samples were imported into Cytobank (https://premium.cytobank.org) (28). For visualization and exploratory analysis, we employed the dimensionality reduction algorithm tSNE-CUDA (t Distributed Stochastic Neighborhood Embedding) on all 44 markers-parameters. Proportional sampling was selected to achieve the highest possible number of total events included in the analysis leading to a total of 9 million total events analyzed from all samples. Perplexity was set to 50 and all other settings (iterations, learning rate and early exaggeration) were set to default/automatic. All related illustrations such as tSNE maps and heatmaps for population densities and marker expression were created in Cytobank. FlowSOM was used for clustering analysis with hierarchical consensus as the clustering method (metaclusters = 50, iterations = 10) in Cytobank (29). For visualization, FlowSOM results were projected on the tSNE map. We also performed differential analysis for specific populations with the algorithm CITRUS in Cytobank. The CITRUS run was configured with Nearest Shrunken Centroid (PAMR) – predictive association model to quantify the abundance of cellular populations with equal sampling, cross-validation folds: 5, minimum cluster size: 5% and false discovery rate: 1%. Ηierarchical clustering and correlation analyses (Spearman correlation coefficient) were performed in R programming environment (Version 4.1.0, https://www.r-project.org/). For these analyses, immune cell frequency data were logged and scaled using the “log10” and “scale” functions of baseR. Complex Heatmap version 2.13.1 (30).

Flow cytometry result analysis was performed using FlowJo™ v10.8 Software (BD Life Sciences, Franklin Lakes, NJ, USA)

### 2.6 MRI acquisition and data analysis

The participants were examined using a Philips Achieva TX 3 Tesla MRI Scanner (Best, the Netherlands) equipped with an eight-channel head coil using the same imaging protocol including: 1) 3D-T1-weighted turbo field echo (TFE) (3D-T1w) sequence (repetition time (TR): 9.9 ms, time echo (TE): 3.7 ms, flip angle 70, voxel size: 0.9 x 0.9 x 0.9 mm, parallel imaging with acceleration factor 2, number of averages: 1, scanning time 359 seconds, sagittal orientation), 2) 3D- turbo spin-echo T2 weighted Fluid-attenuated-inversion recovery (3D-FLAIR) sequences (TR: 9000ms, TE: 600 ms, flip angle 900, inversion time: 2420 ms, number of averages: 2, voxel size: 1.0 x 1.0 x 1.0 mm, scanning time: 711 s, parallel imaging with total acceleration factor 3, sagittal orientation). The 3D-T1w was acquired before and 5 min after an intravenous bolus of 0.1 mmol/kg infusion of gadoterate meglumine (Dotarem) injection (3D-T1wGd); the 3D-FLAIR was acquired immediately after the bolus and 12 min later (3D-FLAIRED). Image analysis was performed independently by two neuroradiologists. Leptomeningeal contrast enhancement foci were identified as hyper-intensities on 3D-FLAIRED and not on 3D-T1wGd sequences; we included in our analysis foci which were confirmed on all three planes (axial, coronal, sagittal) by both raters, as described in one of our recent articles (31).

### 2.7 Statistical analysis

Statistical analysis was performed using GraphPad Prism 9 (version 9.2.0 for Windows, GraphPad Software, San Diego, CA, USA) and the Cytobank platform. For comparisons, normality tests (Kolmogorov–Smirnov test) were first performed, and then Student’s test-test or Mann–Whitney U test was used when appropriate. For comparisons between the different patient groups, a non-parametric Kruskal-Wallis-Test was used and a false discovery rate (FDR) correction method was used for correcting for multiple comparisons between disease subgroups and controls. A p-value < 0.05 was considered statistically significant.

## 3 Results

### 3.1 Expansion of a distinct myeloid signature in TMS patients during a relapse stage

**Figure 1:**
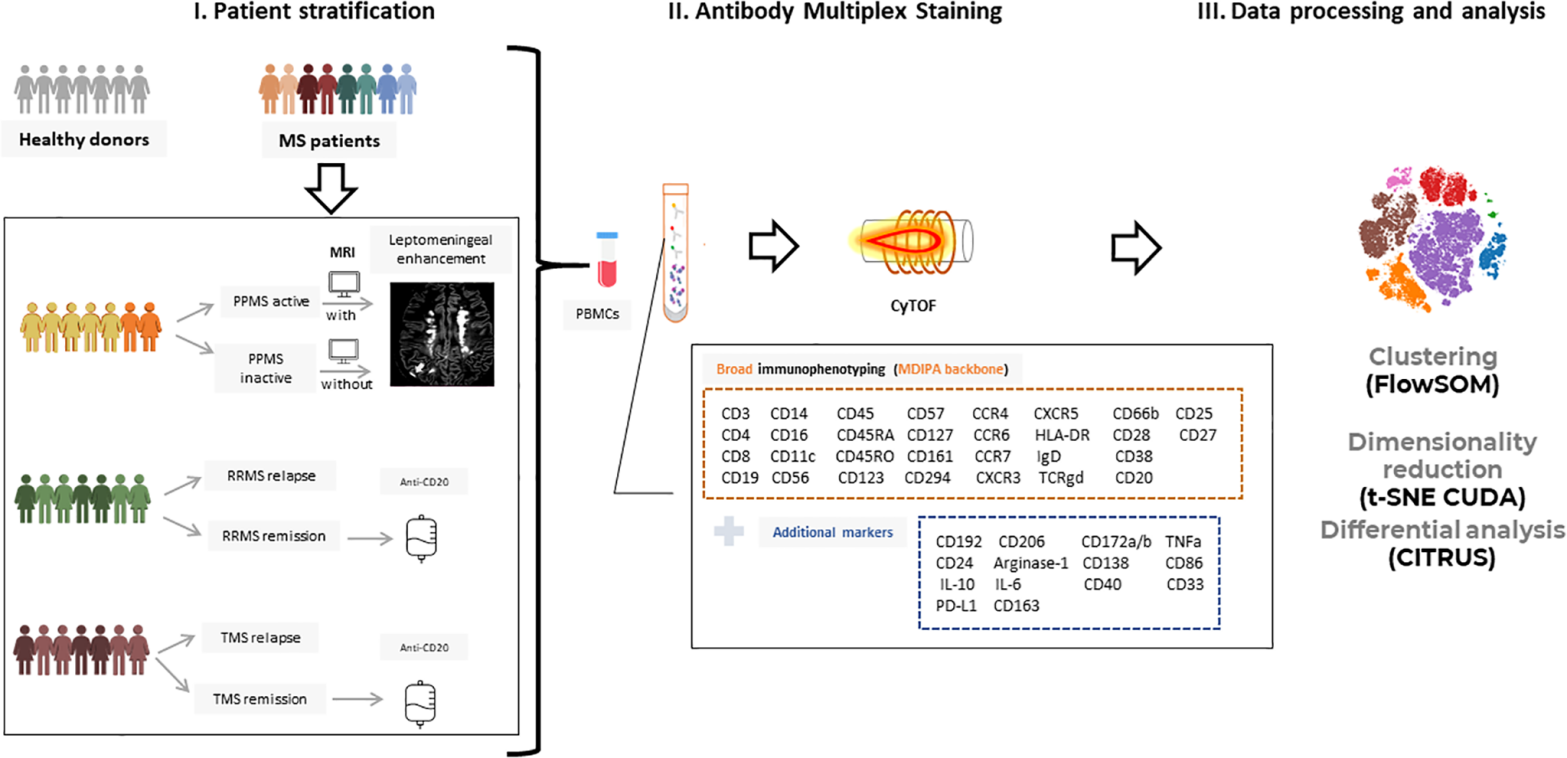
Experimental setup and workflow for mass cytometry analysis. (I) Peripheral blood mononuclear cells (PBMCs) from patients with Multiple Sclerosis (n=32; PPMS=8, RRMS=10, TMS=14) and healthy donors (n=10) were isolated and further stratified in more subgroups based on clinical and radiological criteria. RRMS and TMS were subgrouped based on disease activity in those during a clinical relapse (RRMS; n=5, TMS; n=5) and those under remission (RRMS; n=4, TMS; n=5). PPMS were subgrouped based on the presence of leptomeningeal enhancement on MRI (active PPMS; at least one foci of leptomeningeal inflammation, inactive; without evidence of leptomeningeal inflammation based on MRI criteria). (II) PBMCs were labeled with metal-tagged antibodies against the markers shown in boxes and acquired on a Helios mass cytometer. This multiplex analysis allows broad immunophenotyping as well as deeper analysis in B cell and myeloid subpopulations. (III) Acquired data were analyzed using established analysis pipelines for dimensionality reduction and exploratory data analysis, clustering, and differential analysis. PPMS, Primary Progressive Multiple Sclerosis; RRMS, Relapsing Remitting Multiple Sclerosis; HD, Healthy donors; TMS, Tumefactive Multiple Sclerosis; MRI, magnetic resonance imaging; t-SNE, t-distributed stochastic neighbor embedding; FlowSOM, Flow Self-Organizing Map.

The experimental design of this study is depicted in **Figure 1**. To investigate the peripheral blood immune landscape in MS, we performed CyTOF analysis on PBMCs from HD (n=10) and patients with RRMS (n=10), PPMS (n=8), and TMS (n=14). The patient’s demographic, clinical, and radiological information is summarized in **Table 1**, **Supplementary Table 1**, **2** and **Supplementary Figures 4**, **5**. Peripheral blood analysis with complete blood count (CBC) showed that TMS patients displayed decreased total absolute number of lymphocytes compared to PPMS patients (p=0.015) (**Supplementary Figure 6** and **Table 1**). From TMS patients 2 out of 14 displayed Grade-1 lymphopenia (absolute lymphocyte count 0.8 10^9/L for both), whereas all the others and all RRMS and PPMS patients displayed lymphocytes within normal ranges (1-4.8 10^9/L).

**Figure 2:**
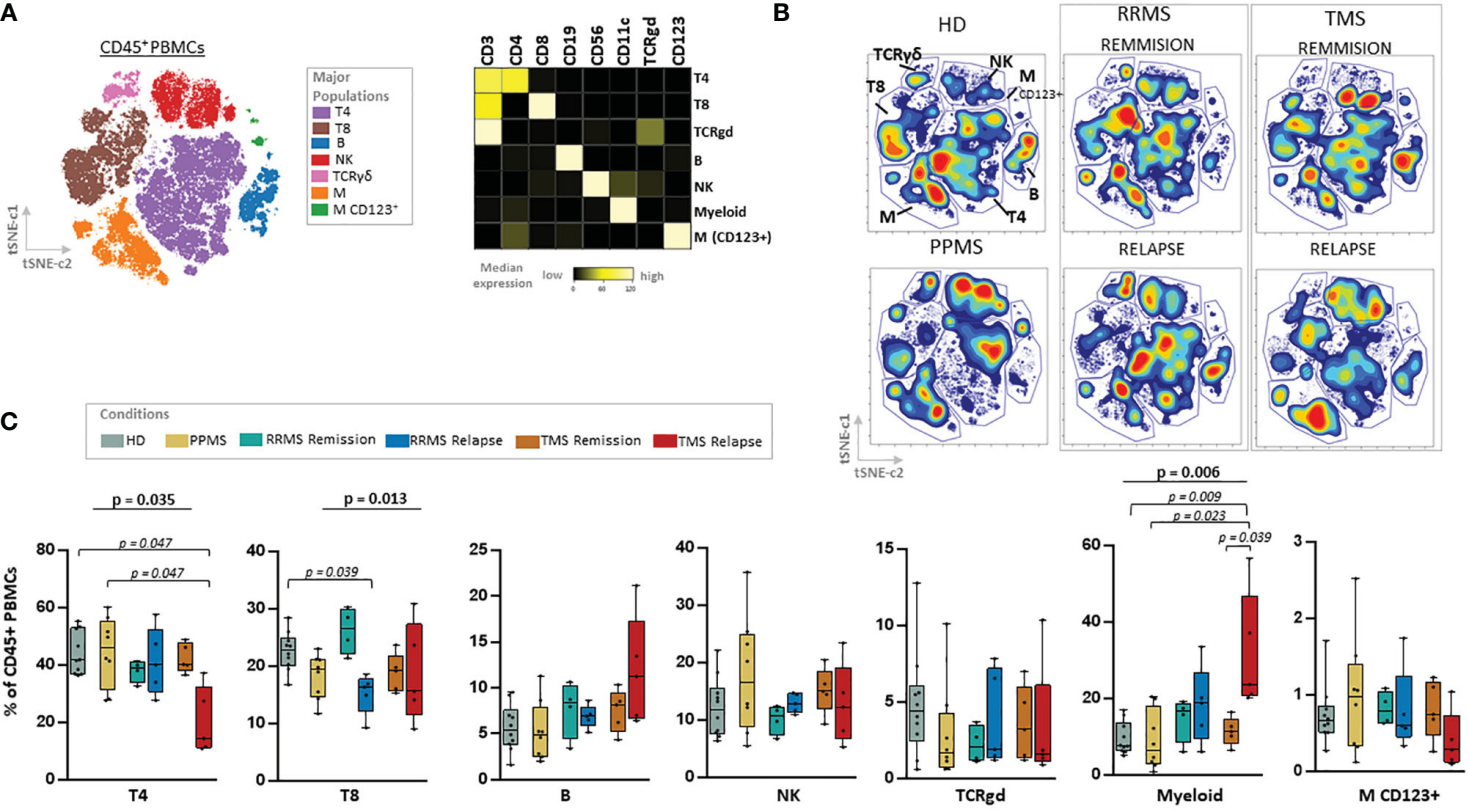
Major cell lineage subsets in peripheral blood of patients with MS during different disease stages and healthy donors. **(A)** Dimensionality reduction with t-SNE CUDA (t Distributed Stochastic Neighborhood Embedding) on all patients and healthy donors tSNE map (left) shows major immune cell subpopulations in the CD45+ compartment (named T4; CD4+ T cells, T8; CD8+ T cells, TCRgd T cells, NK cells, B cells, Myeloid and Dendritic cells). Major PBMC populations were annotated based on the expression of key lineage markers (CD3, CD8, CD19, CD56, CD11c, TCRgd, CD123). **(B)** t-SNE plots from one representative sample from each subgroup (PPMS, RRMS, TMS and HD). RRMS and TMS were further subdivided in those in remission (RRMS; n=4, TMS; n=5) and relapse (RRMS; n=5, TMS; n=5). **(C)** Box plots showing frequency (expressed as % of live singlet CD45+ cells) of each major immune cell subset in peripheral blood mononuclear cells of patient subgroup and healthy donors (n=10). Statistically significant changes are shown in the image (p < 0.05 was considered significant, non-parametric Kruskal Wallis test with correction for multiple comparisons (FDR) was applied). PPMS, Primary Progressive Multiple Sclerosis; RRMS, Relapsing Remitting Multiple Sclerosis; Rem, Remission; Rel, Relapse, HD, Healthy donors; TMS, Tumefactive Multiple Sclerosis; T4; CD4+ T cells, T8; CD8+ T cells, M, Myeloid cells; D, Dendritic cells; NK, natural killer cells; t-SNE, t-distributed stochastic neighbor embedding; FlowSOM, Flow Self-Organizing Map; FDR, false discovery rate.

We used a 44-antibody panel consisting of lineage and activation markers, focused on expanding lymphoid and myeloid subsets (**Supplementary Table 3**). To gain an overview of the data, we performed dimensionality reduction analysis employing the algorithm t-SNE CUDA on all patients (excluding those under anti-CD20 treatment, n=5) and healthy donors. Representative viSNE maps showing the staining pattern of all markers, from one representative sample used in the study, are provided in **Supplementary Figure 7**. We identified seven major immune cell subpopulations (named T4; CD4+ T cells, T8; CD8+ T cells, TCRgd T cells, NK cells, B cells, Myeloid and Dendritic cells), as shown in the t-SNE map (**Figure 2A**). These subpopulations were annotated based on the expression of key lineage markers (CD3, CD8, CD19, CD56, CD11c, TCRgd, CD123) (**Figure 2A**, heatmap). With this analysis, we identified the broad immune landscape profiles in HD and MS patients (**Figures 2B, C**). In particular, the generated t-SNE maps showed that PPMS patients display similarities with HD (except for a relative expansion of NK cells). In contrast, RRMS patients share features with TMS patients (**Figures 2B, C**). We did not measure significant differences in major lymphoid subsets (such as T4, T8, B, NK TCRgd), when comparing HD with PPMS, TMS and RRMS (**Supplementary Figure 7**). However, we observed a trend towards higher frequencies of myeloid cells in TMS (HD versus TMS in relapse; adjusted p=0.07) (**Supplementary Figure 8**).

we subgrouped RRMS stages of remission or relapse. TMS patients in relapse were mostly differentiated from other disease subgroups due to the expansion of myeloid cells (HD versus TMS in relapse; adjusted p= 0.009) and reduction of CD4+ T cells (HD versus TMS in relapse; adjusted p= 0.047). RRMS patients in relapse displayed, in contrast to TMS patients in relapse, a reduction of CD8+ T cells (Kruskal Wallis H test, p= 0.013, HD versus RRMS in relapse; adjusted p= 0.039) (**Figure 2C**).

**Figure 3:**
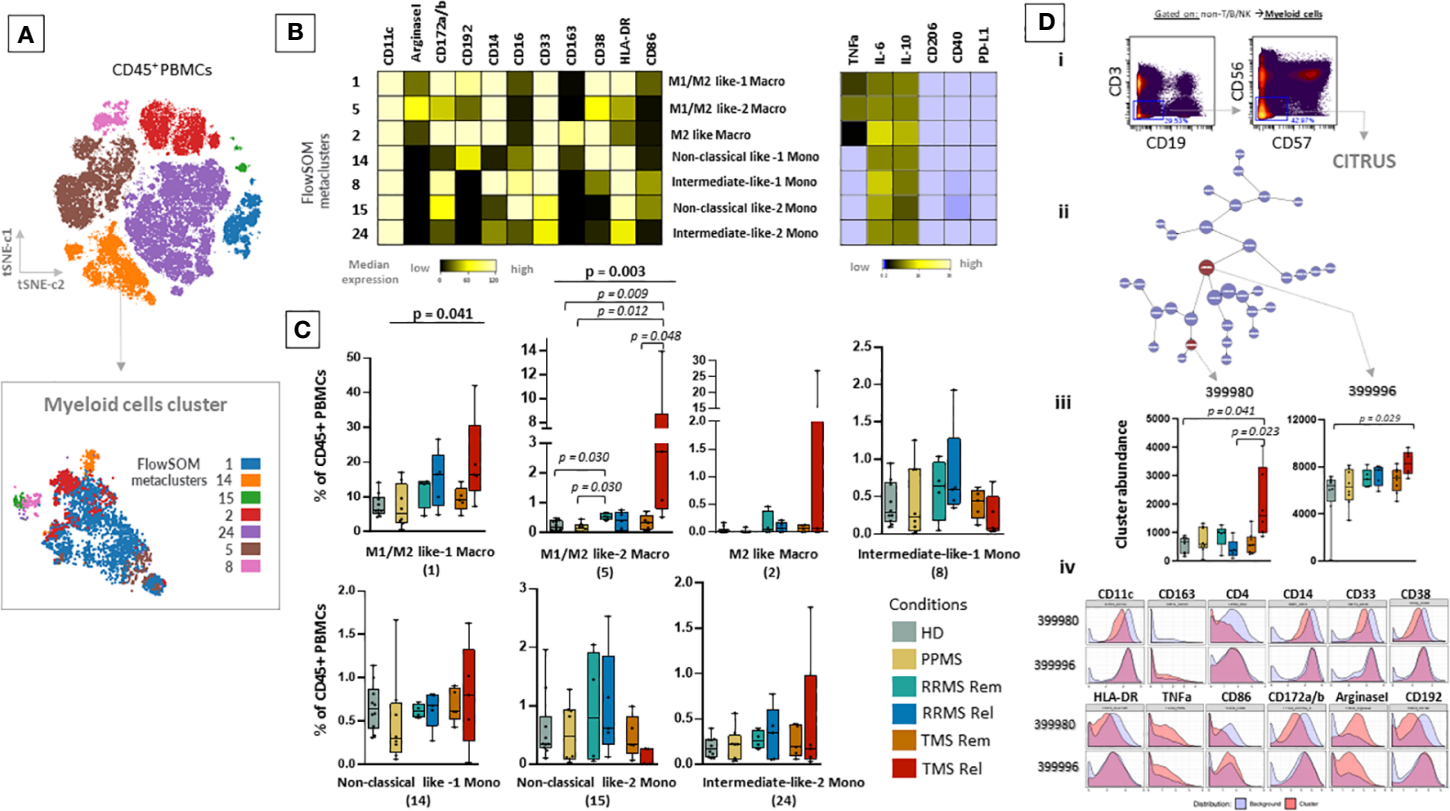
Expansion of a myeloid specific signature in TMS patients during relapse. **(A)** FlowSOM resulted metaclusters in myeloid cells projected on the tSNE map (a representative example from one healthy donor is shown here). **(B)** Heatmap for key markers to identify to characterize metaclusters. A tentative biological name was assigned to each metacluster based on the most abundant myeloid markers expressed by each cluster. **(C)** MS patients stratified in different disease subgroups (PPMS; n=8, RRMS; n=9, TMS; n=10). RRMS and TMS were further subdivided in those in remission (RRMS; n=4, TMS; n=5) and relapse (RRMS; n=4, TMS; n=5). Box plots showing the frequency (expressed as % of CD45+ cells) of each myeloid cell metacluster in peripheral blood mononuclear cells of patient subgroups and healthy donors (n=10). Statistically significant changes are shown in the image (p < 0.05 was considered significant, non-parametric Kruskal Wallis test with correction for multiple comparisons (FDR) was applied). **(D, i)** Gating strategy for cells belonging to the Myeloid cell compartment (non-T/B/NK) seeded in the CITRUS algorithm. CITRUS results include the features **(D, ii)**, abundance of clusters that differentiate groups in comparison **(D, iii)** and histograms of the expression of markers to identify the immune subsets that these clusters represent **(D, iv)**. PPMS, Primary Progressive Multiple Sclerosis; RRMS, Relapsing Remitting Multiple Sclerosis; Rem, Remission; Rel, Relapse; TMS, Tumefactive Multiple Sclerosis; HD, Healthy donors; t-SNE, t-distributed stochastic neighbor embedding; FlowSOM, Flow Self-Organizing Map; CCR2 or CD192, C-C chemokine receptor type 2, CD172a/b or SIRPa/b, signal-regulatory protein alpha/beta; PD-L1, Programmed death-ligand 1; TNF-α, tumor necrosis factor alpha; IL-10, Interleukin 10; FDR, false discovery rate.

To gain a deeper understanding of the specific subsets present in the peripheral blood of MS patients, we performed a clustering analysis (50 metaclusters, FlowSOM algorithm) projected on the t-SNE map. The expression of major lineage markers as well as the relative frequencies of these metaclusters are shown in **Supplementary Figure 9**. We identified seven metaclusters belonging to the myeloid cell lineage that were annotated based on key lineage markers as well as endogenous cytokine production (**Figures 3A, B**). TMS patients during relapse are characterized by the expansion of intermediately activated macrophages with both features of M1 (pro-inflammatory) and M2 (alternatively activated, anti-inflammatory) cells, referred here as M1/M2 like-1 cells (HD versus TMS in relapse; adjusted p= 0.009, TMS in relapse versus TMS in remission; adjusted p=0,048) (**Figure 3C**). These presented high expression of CD11c, Arginase-1, CD14, CD38 and CD33, intermediate levels of CD172a/b, CD192 and low levels of CD16, CD86, CD163 (**Figures 3A, B**). Regarding cytokine expression, the expanded M1/M2 like-1 population in TMS patients expressed more TNF-a than other myeloid subsets. (**Figure 3B**). This specific myeloid cell population (metacluster – 5) was also found to expand in TMS patients when patients in relapse and remission were grouped together (**Supplementary Figure 10**).

To confirm these results, we also performed differential analysis with the CITRUS algorithm on the myeloid cells as shown in **Figure 3D**. This analysis identified two clusters enriched in TMS patients during relapse that differentiated them not only from TMS in remission but also from RRMS during relapse or remission and PPMS compared to HD. These cell clusters corresponded to a subset of unique intermediately activated macrophages with an increased expression of Arginase-1 and TNF-a and low expression of markers indicative of efficient antigen presentation capacity (HLA-DR and CD86) (**Figure 3D**). Collectively, our findings suggest that MS patients present distinct peripheral blood immune profiles and more specifically highlight that, within this heterogeneity, TMS patients are characterized by a unique cellular blueprint in the myeloid compartment.

### 3.2 Immune profiling with conventional flow cytometry and serum neurofilament confirm TMS-specific myeloid cell signature during active disease stages

To confirm this myeloid signature by an independent method in the same population, we tested PBMCs from the same cohort with conventional flow cytometry for the presence of myeloid cells. We stained the PBMC from 5 healthy individuals, 4 RRMS patients in remission, 4 RRMS patients in relapse, 5 TMS in relapse and 5 TMS patients in remission with markers of myeloid cell lineage that belong to the specific cell signature that came out from our clustering strategy and CITRUS algorithm (CD11c+ CD14+ cells). Such analysis confirmed that TMS patients in relapse are characterized by the expansion of myeloid cells that are CD11c, and CD14 positive (negative in CD3, CD19, CD56). Importantly, as shown in the **Supplementary Figure 11**, this analysis revealed that myeloid cells can be divided in two subpopulations based on their forward scatter (FSC) properties (FSChigh and FSClow). TMS in relapse are specifically characterized by an expanded fraction of FSChigh myeloid cells that are CD11c+, CD14+.

Serum neurofilament light chain (NfL) is a blood biomarker in MS specific for neuronal injury or neurodegeneration. Patients with relapse or radiologic activity display significantly higher serum NFL levels than those in remission, and significantly, effective disease-modifying treatments reduce NFL levels (32, 33). To assess the association of the myeloid cell signature with neuroaxonal damage, NfL levels were determined in our cohort. TMS patients in relapse displayed increased serum NfL age-adjusted scores compared to TMS in remission (p = 0.0012). In TMS patients in relapse, NfL levels showed a good correlation with the frequency of the myeloid cell population (**Supplementary Figure 12**).

### 3.3 TMS patients in relapse display unique phenotypic alterations in the CD4+ T cell and memory B cell lymphoid compartment

**Figure 4:**
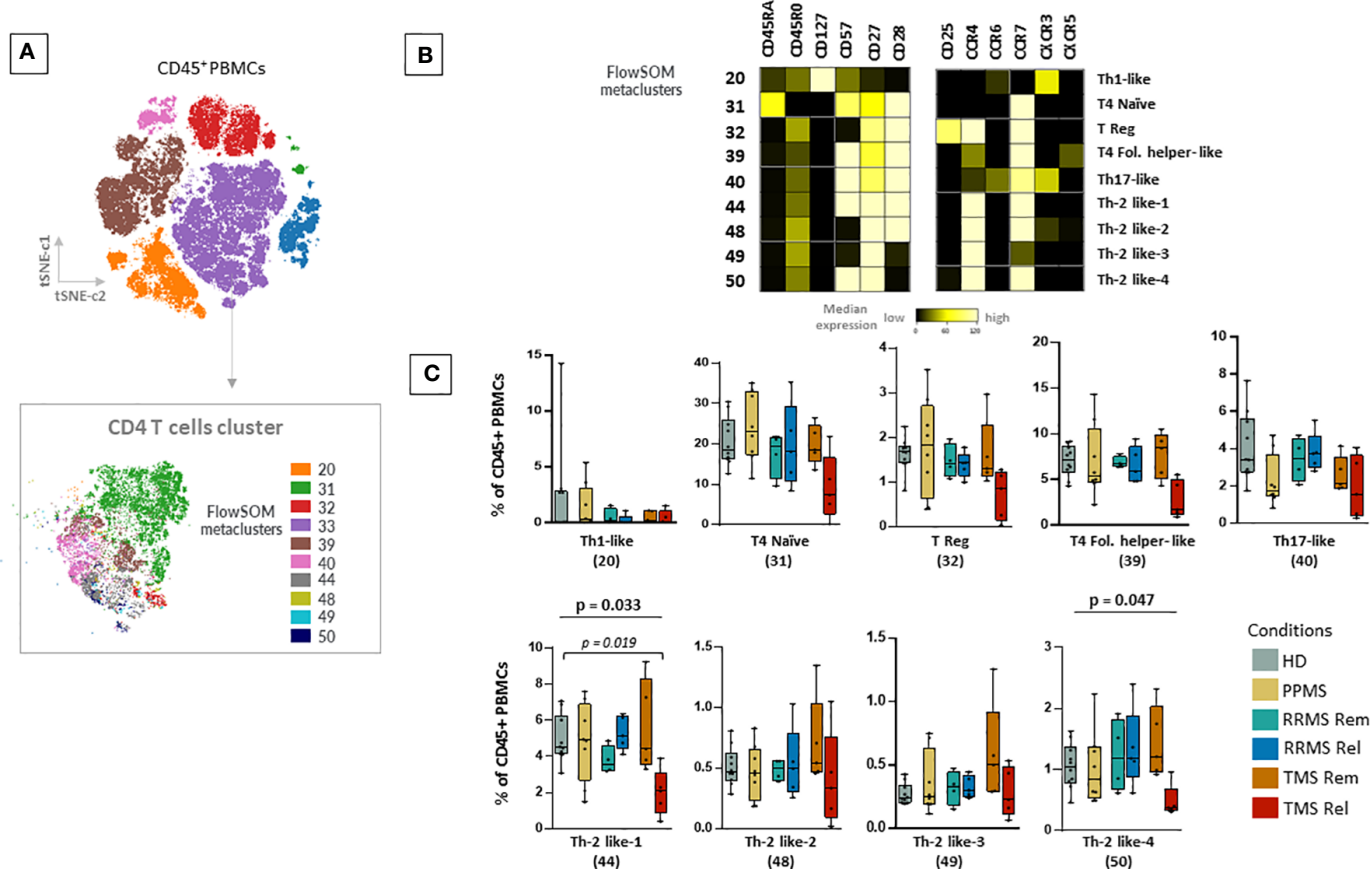
Selective CD4+ T cell lymphopenia in TMS patients during relapse. **(A)** FlowSOM resulted metaclusters in CD4+ T helper cells projected on the tSNE map (a representative example from one healthy donor is shown here). **(B)** Heatmap for key markers to identify to characterize metaclusters. A tentative biological name was assigned to each metacluster based on the most abundant myeloid markers expressed by each cluster. **(C)** MS patients stratified in different disease subgroups (PPMS; n=8, RRMS; n=9, TMS; n=10). RRMS and TMS were further subdivided in those in remission (RRMS; n=4, TMS; n=5) and relapse (RRMS; n=4, TMS; n=5). Box plots showing the frequency (expressed as % of CD45+ cells) of each CD4+ T cell metacluster in peripheral blood mononuclear cells of patient subgroups and healthy donors (n=10). Each dot represents the value of each sample. Statistically significant changes are shown in the image (p < 0.05 was considered significant, non-parametric Kruskal Wallis test with correction for multiple comparisons (FDR) was applied, p<0.05, p<0.01). PPMS, Primary Progressive Multiple Sclerosis; RRMS, Relapsing-Remitting Multiple Sclerosis; Rem, Remission; Rel, Relapse; TMS, Tumefactive Multiple Sclerosis; HD, Healthy donors; IQR, interquartile range; t-SNE, t-distributed stochastic neighbor embedding; FlowSOM, Flow Self-Organizing Map; Eff., Effector cell population; T4; CD4+ T cell, Fol., Follicular T helper cells, Th1-like, T helper 1-like cells, Th-2 like; T helper 2 -like cells; CCR, CC chemokine receptors; CXCR, CXC chemokine receptor; FDR, false discovery rate.

Next, we aimed to explore other immune cell families such as T cells and B cells. With the FlowSOM algorithm, we identified and annotated 9 subpopulations of CD4+ T cells (**Figures 4A, B**). In TMS patients during relapse, compared to those in remission, we observed a selective CD4+ T cell lymphopenia, with a lower abundance of two T cell subsets with Th2-like cell properties, referred here as Th-2 like-1 (HD versus TMS in relapse; adjusted p= 0.019) and T4 Th-2 like-4 (Kruskal Wallis H test, p= 0.047) (**Figure 4C**). These cell subsets were mainly CD45RA-, CD45RO+, CXCR3-, CXCR5-, CCR6-, CCR4+, CCR7+, CD127-, CD57+ and CD27+ (**Figures 4A, B**). Regarding the CD8+ T cell compartment, as well as TCRgd and CD123 dendritic cells, we did not find significant alterations (**Supplementary Figure 13** and data not shown). Concerning B cells, we identified 6 metaclusters (**Supplementary Figures 14A–C**). We found one metacluster with a significantly different abundance between TMS patients and PPMS (PPMS versus TMS in relapse; adjusted p=0.047). This cell subset corresponds to a double negative B cell (CD19+CD20+CD27- IgD-HLA-DR+CD38-) subset that moderately expresses migration and activation markers (CXCR3, CCR7, CD40) (**Supplementary Figure 14B**).

### 3.4 Specific drug-associated phenotypic alterations in myeloid cells

**Figure 5:**
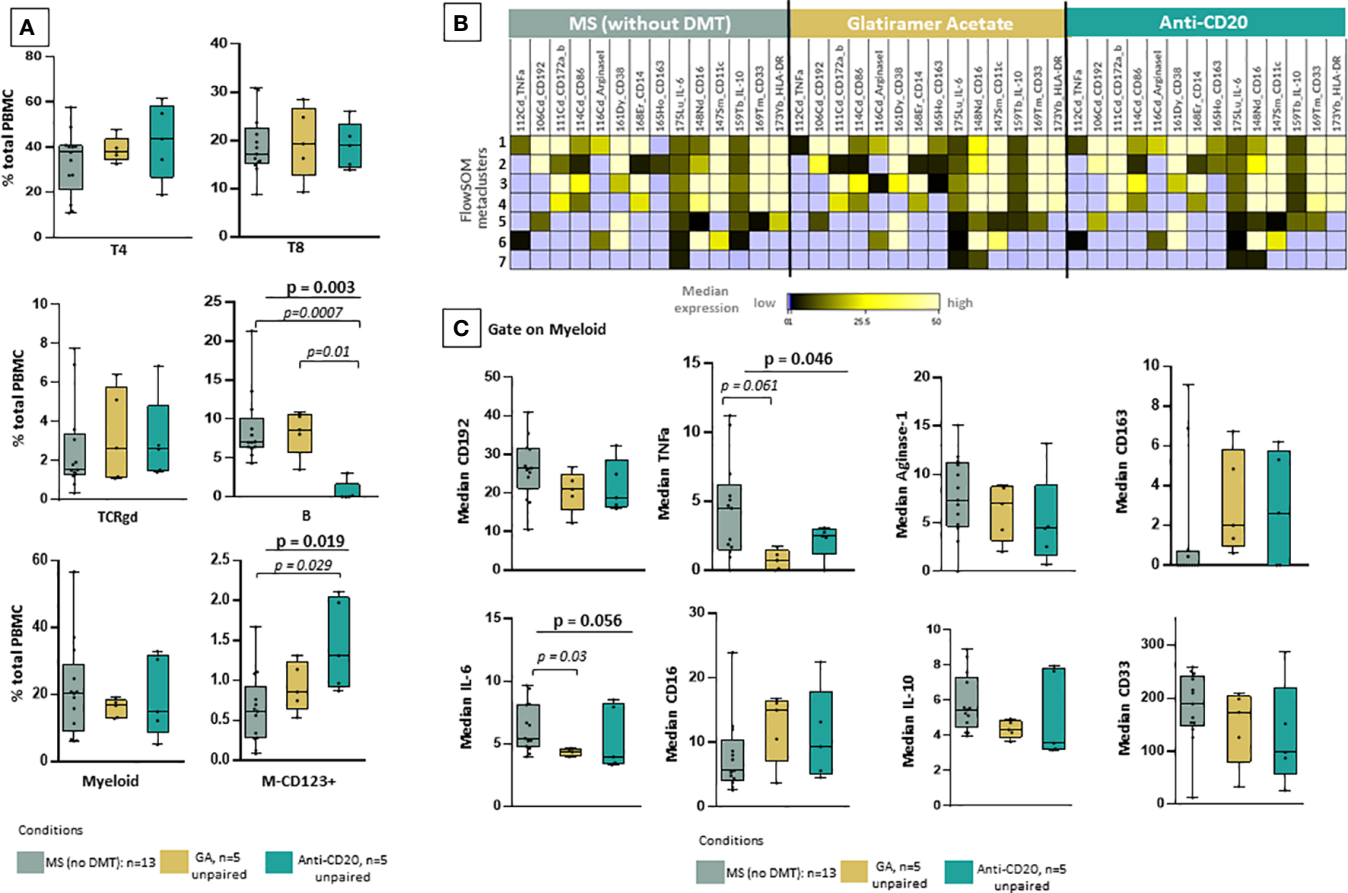
Reshaping of myeloid signature population under glatiramer acetate and anti-CD20 therapy. **(A)** Box plots showing the frequency (expressed as % of CD45+ cells) of each major cell-lineage cluster in peripheral blood mononuclear cells of MS patients divided in three groups; those under no anti-DMT therapy (n=13, both TMS and RRMS are included, PPMS excluded), those under anti-CD20 treatments (n=5, both TMS and RRMS are included) and those under glatiramer acetate (n=5, both TMS and RRMS are included). **(B)** FlowSOM identified metaclusters in myeloid cells and heatmap showing, for each of the metaclusters generated, in the three groups of interest (MS under no DMT, MS under glatiramer acetate, MS under anti-CD20), the average intensity of each myeloid cell related marker (stage or activation marker). Higher average expression of each marker is indicated with a green-yellow color, and lower expression in black. Absence of expression is depicted with blue. **(C)** Box plots showing the median expression of each marker on total myeloid cells in peripheral blood mononuclear cells of patient subgroups (MS under no DMT, MS under glatiramer acetate, MS under anti-CD20). **(D)** tSNE plots of one TMS patient during relapse and after anti-CD20 treatment. The expression levels of indicative markers are shown in plots. p < 0.05 was considered significant, non-parametric Kruskal Wallis test with correction for multiple comparisons (FDR) was applied). FDR, false discovery rate; TMS, Tumefactive Multiple Sclerosis; DMT, disease modifying therapy; FlowSOM, Flow Self-Organizing Map; CCR2 or CD192, C-C chemokine receptor type 2TNF-α, tumor necrosis factor alpha, IL-10; Interleukin 10, IL-6; Interleukin 6.

To further investigate the clinical importance of our findings, we examined phenotypic alterations in patients under key treatment modalities. Using a similar analytical approach (dimensionality reduction and clustering analysis), we investigated whether there are any drug-related effects in myeloid subsets. To this aim, we grouped the patient cohort in drug naïve MS patients during sample collection (including RRMS and TMS, n=13) and those under anti-CD20 treatment (n=5) and glatiramer acetate (n=5) treatment. One major limitation is that this analysis did not include longitudinal paired samples during different disease stages. Regarding major cell subsets, our analysis confirmed that anti-CD20 treatment significantly reduces all B cell subsets (**Figure 5A**). Most importantly, our analysis revealed that predominantly glatiramer acetate and to a lesser extent anti-CD20 treatment associates with the remodeling of myeloid subsets (**Figure 5B**). The new myeloid cell meta cluster 1 corresponds to M1/M2 like cells predominantly found in TMS during relapse. Under treatment, a distinct phenotype with more M2-like features (reduction of TNF-a and upregulation of CD163) was evident (**Figure 5B**). Of note, we measured reduced levels of TNF-a and IL-6 in myeloid cells from patients under glatiramer acetate treatment (**Figure 5C**). Most importantly, we further explored the immune landscape before and after B cell depletion in one TMS patient (**Supplementary Figure 5**). To this aim, we analyzed data from one TMS patient in whom we isolated PBMCs at disease exacerbation and after anti-CD20 treatment. We performed t-SNE analysis with PBMC samples that were taken before (during relapse) and after successful treatment (during remission) (**Supplementary Figure 15**). Here, we can see the different distribution of myeloid metaclusters before and after anti-CD20, as well as the overall reduction of myeloid cells. Interestingly, a certain area of myeloid cells in the t-SNE map indicated that a fraction of myeloid cells with activated phenotype remain unaffected (**Supplementary Figure 15**). Nevertheless, longitudinal observation of these cell subsets, as well as validation in a larger cohort, could give more insights towards their role in disease progression.

### 3.5 PPMS patients with leptomeningeal enhancement display a specific NK cell signature in the peripheral blood

**Figure 6:**
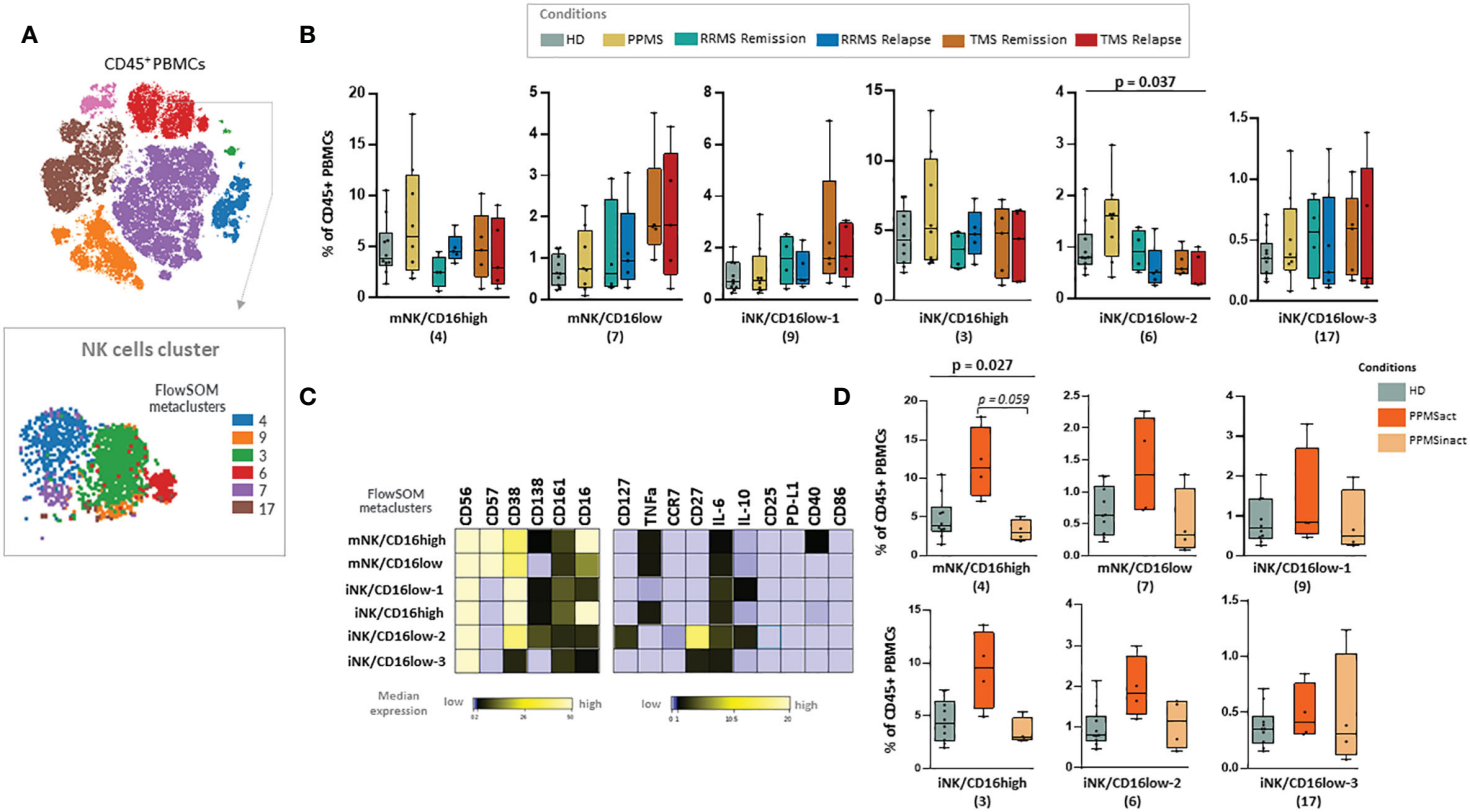
Natural killer (NK) cell populations expanded in PPMS patients characterised by the presence of leptomeningeal enhancement in MRI. **(A)** FlowSOM identified metaclusters in NK cells (a representative example from one healthy donor is shown here). **(B)** Box plots showing the frequency (expressed as % of CD45+ cells) of each NK cell metacluster in peripheral blood mononuclear cells of patient subgroups and healthy donors (n=10). MS patients stratified in different disease subgroups (PPMS; n=9, RRMS; n=9, TMS; n=10). RRMS and TMS were further subdivided in those in remission (RRMS; n=4, TMS; n=5) and relapse (RRMS; n=4, TMS; n=5). A tentative biological name was assigned to each metacluster based on marker expression, shown in the heatmap **(C)**. **(D)** Box plots showing the frequency (expressed as % of CD45+ cells) of each NK cell metacluster in peripheral blood mononuclear cells of PPMS patients subgrouped in those characterised by the presence of leptomeningeal enhancement (PPMSact) and those without (PPMSinact), as assessed by novel MRI techniques (shown in material and methods). p < 0,05 was considered significant, non-parametric Kruskal Wallis test with correction for multiple comparisons (FDR) was applied, * p<0,05). PPMS, Primary Progressive Multiple Sclerosis; act, active; inact, inactive; RRMS, Relapsing Remitting Multiple Sclerosis; Rem, Remission, Rel, Relapse; TMS, Tumefactive Multiple Sclerosis; HD, Healthy donors; IQR, interquartile range; t-SNE, t-distributed stochastic neighbor embedding; FlowSOM, Flow Self-Organizing Map; PD-L1, Programmed death-ligand 1; TNF-α, tumor necrosis factor alpha; IL-10, Interleukin 10; IL-6, Interleukin 6; CCR, CC chemokine receptors; FDR, false discovery rate; i, immature; m, mature.

PPMS patients did not display significant differences compared to the other disease subgroups or HD. We were able to observe a non-statistically significant trend towards enrichment in NK cell subsets and especially the cluster NK-6 (or named iNK/CD16low-2) that corresponds to immature NK cells (CD56+++, CD38++, CD27++, CD16+, CD57-) (Kruskal Wallis H test, p= 0,037) (**Figures 6A–C**). PPMS constitutes an heterogenous group that can be further divided into two groups based on the presence of leptomeningeal enhancement on MRI that was performed at the same time as sample collection. Among PPMS patients, 4 out of 8 displayed leptomeningeal enhancement on MRI (**Supplementary Table 3** and **Supplementary Figure 5**). We found an expansion of one population of NK cells with a phenotype suggestive of terminally differentiated mature NK cells (NK-4 or named mNK/CD16high, characterized by CD56+, CD57+, CD16+, CD38+) in PPMS patients with leptomeningeal enhancement (active PPMS) compared to those without (inactive PPMS) (Kruskal Wallis H test, p= 0.027, PPMS active versus inactive; adjusted p-value = p = 0.059) (**Figures 6C, D**). Moreover, a trend towards expanding another NK cell population with highly cytotoxic properties (NK-3 or named iNK/CD16high, characterized by CD56+, CD38+, CD16+, CD57-) was found in active PPMS patients, albeit it did not reach statistical significance (**Figures 6C, D**).

### 3.6 A Th-2 like-DN B cell-M1/M2myeloid axis in peripheral blood characterizes TMS patients in relapse

**Figure 7:**
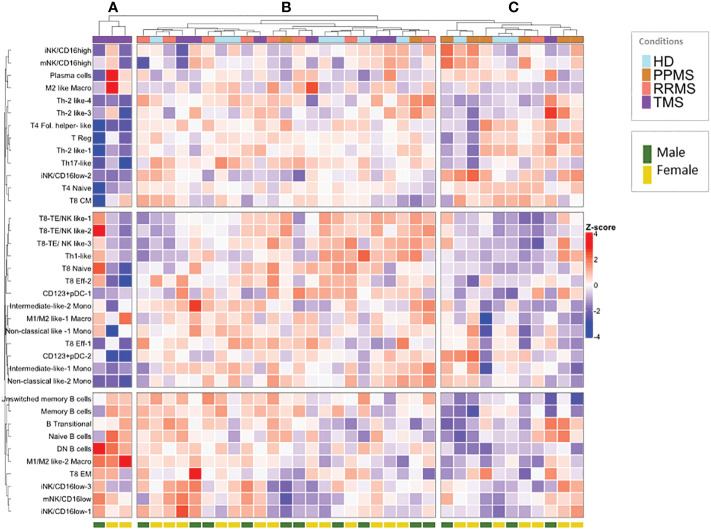
Hierarchical clustering of normalized peripheral blood immune subset frequencies in HD and MS patients. Hierarchical clustering **(A–C)** of normalized immune subset frequencies (% of total CD45+ cells) in peripheral mononuclear cells from all patients (n=27; PPMS=8, RRMS=9, TMS=10) and healthy donors (HD; n=10). Gender (male, female) from healthy donors and patients is also indicated in the lower part of the heatmap.

To identify patterns in the peripheral blood immune profiles of MS patients, we implemented hierarchical clustering of immune subsets (% of total CD45+ cells) of all samples in both patients (n=27; PPMS=8, RRMS=9, TMS=10, anti-CD20 treated patients excluded) and HD controls (n=10) (**Figure 7**). Three major patterns were apparent in this analysis. Firstly, three TMS patients clustered together and were mainly characterized by high M1/M2 like Macro and DN B cell levels and low T4 levels (**Figure 7**, branch A). Secondly, the rest of TMS patients clustered together with the rest of the samples in the most heterogeneous tree branch (**Figure 7**, branch B), which is largely spared from PPMS patients. TMS patients in this branch differ from patients of the same group mainly in T cell characteristics. Finally, a third branch (**Figure 7**, branch C) presented almost the reverted immune profile compared to branch A, and consisted predominantly of PPMS patients and HD controls No gender bias was observed in this analysis (**Figure 7**).

**Figure 8:**
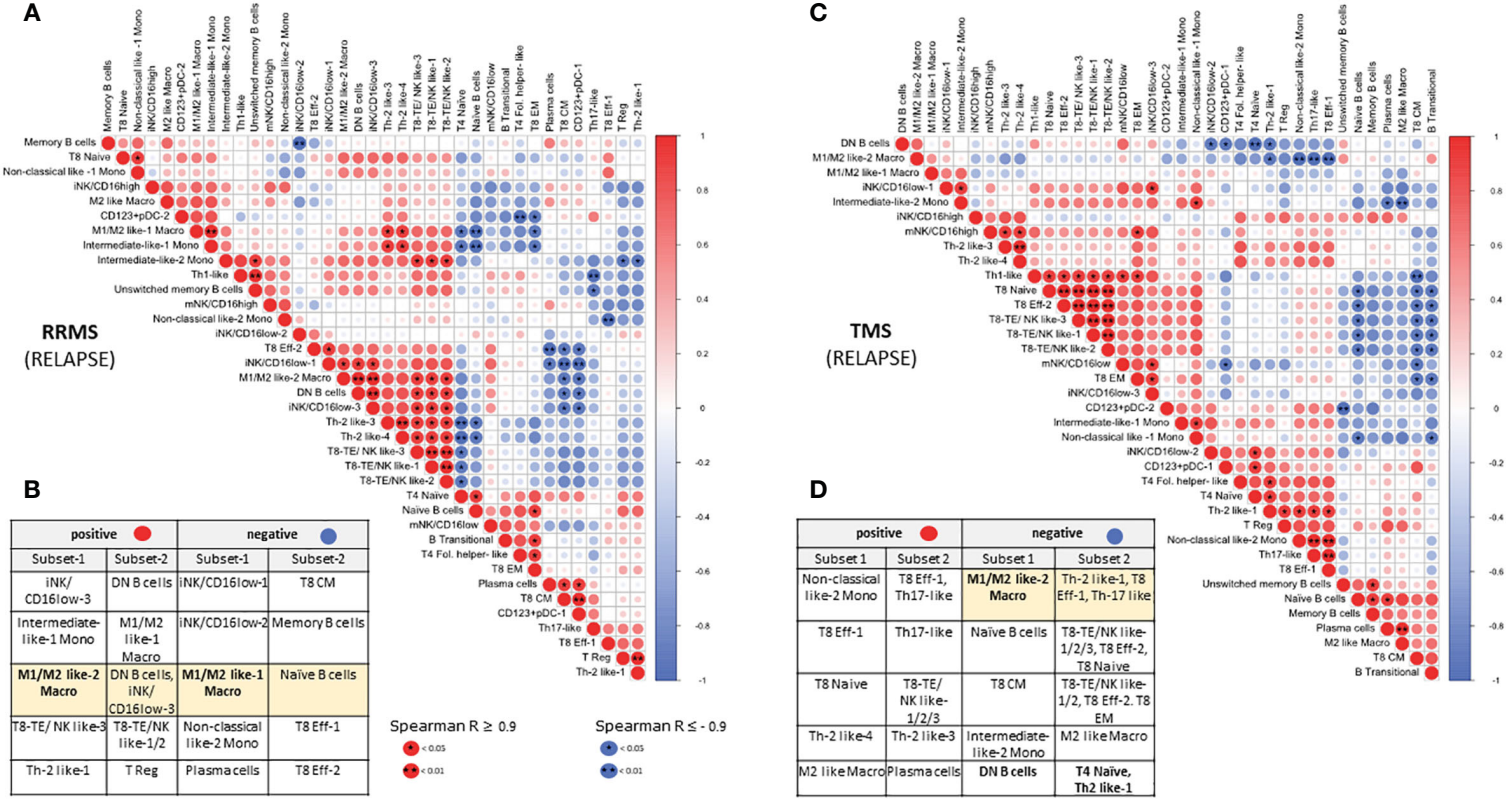
Correlation matrix of immune subsets in relapsing MS (RRMS and TMS) patients. Relationships between immune subsets in RRMS during relapse (matrix A and table B) and TMS patients during relapse (matrix C and table D). In the matrices, **(A, C)** correlations are color-indicated (red for positive, blue for negative). Selected top significant positive correlations with a Spearman *R* > 0.9 and negative *R* < -0.9 are shown in tables, **(B, D)** *p <0.05, ** p<0.01.

Our hierarchical clustering analysis suggested that TMS patients harbor distinct immunological profiles as well as some similarities with other types of MS. To gain further insights into possible biological correlations between RRMS and TMS patients in relapse, we complemented this analysis with a correlation matrix (**Figure 8**). We marked several powerful correlations that were mostly distinct in these two conditions. Our analysis confirmed that the M1/M2 like-2 macrophage (a key cellular signature that we identified in TMS patients) negatively correlates with CD4+ T effector subsets (such as Th2-like) only in TMS patients. Moreover, we found that this myeloid subset positively correlates with DN B cells and negatively correlates with naïve B cells only in RRMS patients. We also noted that double-negative DN B cells also negatively correlate with CD4 Th2-like cells. Considering these findings, we conclude that disease-specific inflammatory macrophage- Th2-like – DN B cell axis could be a prominent regulatory network present in the early TMS patients with pathogenetic potential. Another difference that hints at distinct immunological patterns in these two MS types are the correlations found for Th1 cells. In relapsing RRMS patients, Th-1 cells positively correlate with unswitched memory B cells and negatively correlate with Th-17 cells. On the contrary, Th-1 cells in relapsing TMS patients correlate with CD8 effector subsets with cytotoxic potential (including NK-like subsets) (**Figure 8**). Further functional experiments are needed to provide a biological foundation for these observations. A schematic representation of cell types differentially expressed in peripheral blood as revealed by broad immunophenotyping with CyTOF analysis, as well as their inter-correlations, in active RRMS, active TMS and active PPMS patients is provided in **Supplementary Figure 16**.

## 4 Discussion

In the present study, we provide the first evidence for the role of extensive immune profiling by multiplexed single-cell mass cytometry coupled with computational algorithms to capture phenotypic alterations in specific innate and adaptive immune cell populations in PBMC samples of RRMS, TMS and PPMS patients. CyTOF analysis has not been performed previously in PBMCs from PPMS (less frequent MS form) and TMS (rare MS variant) patients. Our results clearly show that, based on peripheral blood immunophenotyping, TMS patients display similarities with RRMS patients, whereas PPMS have minimal differences from healthy individuals and other MS patients. In-depth analysis of different clinical stages of patients revealed a unique signature that features sub-populations of myeloid cells in the peripheral blood of patients with TMS, especially during relapse, characterized by the expression of CD14, CD33, CD11c, CCR2, CD172, TNF-a, CD38 Arginase-1 and HLA-DR. Finally, patients with PPMS with signs of active inflammation in the CNS, as evident by leptomeningeal enhancement by novel MRI techniques, displayed a cell population of NK cells with highly cytolytic capacity and features of terminal differentiation status.

Immunopathologic studies have revealed massive aggregation of macrophages/microglial cells in tissue biopsies from TMS patients. CNS-specific factors previously observed in TMS patients include activated microglia expressing TMEM119, deregulated pathway of hypoxia-inducible factor-1α (HIF-1α) in Balo’s disease, deregulated S1P receptors in astrocytes after fingolimod cessation, and plasma cells in natalizumab-treated patients (34–36). Information from the periphery is only based on case reports, and persistent selected peripheral lymphopenia (CD8 or CD4+ T cells) has been described in a few cases (37). Regarding neuropathologic evidence, there are no specific findings and hallmarks differentiating TMS from classic MS. Differences are mainly quantitative, whereas qualitative aspects of disease pathogenesis have not been addressed till today. On the other hand, PPMS, a disease not involving relapses/remissions but gradual clinical deterioration, is characterized by a more compartmentalized inflammation behind a closed blood-brain barrier. In general, classical active white matter lesions and total degree of inflammation are milder in PPMS compared to RRMS (38, 39). Therefore, we hypothesized that extensive peripheral blood immunophenotyping in patients with demyelinating diseases in the spectrum of MS, with cardinal immunopathological differences between them (TMS, PPMS), could serve as a tool to identify new biomarkers and new pathogenetic aspects reflecting specific disease traits.

In the lymphoid cell compartment of drug naïve patients with MS, we observed in RRMS during relapse a CD8+ T cell naïve lymphopenia and in TMS during relapse a CD4+ T cell lymphopenia. As established, CD8 tissue-resident memory cells are the major cell type in acute MS lesions (40). CD8+ T cells are abundant and clonally expanded in MS lesions, suggesting local antigen recognition (41–44). Regarding TMS patients, comparative studies with RRMS have never been performed. A recent large-scale CyTOF analysis reported phenotypic, but no compositional changes in blood leukocytes from MS patients compared with controls. This study identified an MS-specific expansion of CD4+ T cells producing cytotoxic granulocyte-macrophage colony-stimulating factor (GM-CSF) and expressing cell surface CXCR4 that was absent in controls (24). Another study analyzed PBMCs isolated from healthy donors and drug-naïve patients with early MS using CyTOF analysis. An increased abundance of CCR7+ and IL-6+ T cells was detected in PBMCs from early MS, whereas the population of NFAT1hiT-bethiCD4+ T cells was decreased. Changes in the subset composition of the monocytes were not observed in this study in early MS patients (19). Patients under the active disease stage close to relapse and patients with Tumefactive MS were not included, further pointing to the distinct features displayed by TMS patients revealed in our study.

In TMS patients in relapse we found a mild increase in DN B cells (CD19+CD20+IgD-CD27-CD38-). Recent studies have investigated B cell heterogeneity in MS with CyTOF analysis. Couloume et al. recently found an increased abundance of a T-bet-expressing B cell subset (CD19, CD27, CD38, T-bet, CXCR3, CCR4, and Ki67) in the blood of patients with aggressive MS. Nevertheless, it is unclear whether patients with TMS were included in this study (45). Marsh-Wakefield et al. (46) used a focused-cell-focused mass cytometry panel to compare peripheral IgG3+ IgD- B cells of MS patients at inactive or active stages of the disease. Previous molecular and flow cytometry studies have shown that double‐negative (DN; CD27-, IgD-) B cells are abnormally elevated in MS and NMOSD patients with active forms of the disease, a finding that is in agreement with our results (47, 48). In our study, we provide a more detailed phenotypic description of such cells, finding that they belong to the CXCR5- subgroup that is thought to constitute the extrafollicular DN2 subset in active Systemic lupus erythematosus (SLE) (49). There is also evidence that these “DN2” cells differentiated into autoantibody-producing plasma cells driven by TLR7, which led to their characterization as extra-follicular B cells responding to innate stimuli (50). Nevertheless, it is currently unknown whether the origin of these cells in TMS patients is derived from an extrafollicular maturation pathway or a senescent process leading to aged/exhausted B cell expansion.

Our in-depth analysis using 44 markers showed that TMS cases are enriched in a myeloid cell cluster that is defined by CD14, CD33, CD11C, CD192, CD172/SIRPa/b, Arginase-1, TNF-a and CD38. Increased abundance of a myeloid cell subset in peripheral blood of early MS patients with a phenotype corresponding to classical monocytes with pro-inflammatory markers (markers associated with M1 macrophage polarization, CD86, CD64, CD32), regulatory markers (markers related to M2 macrophage polarization, CD206, CD209, PD- L1) and a high expression of S100A9 have been recently shown by Colloume et al. (45). The majority of activated macrophages in active MS lesions were shown to display a mixed pro-inflammatory and regulatory status. iNOS/CD206 double positive macrophages were detected in all chronic active MS lesions examined with a higher frequency in the MS lesion center (51). The co-expression of both regulatory and inflammatory markers could reflect myeloid plasticity and a transitional process from highly pro-inflammatory cells to myeloid cells with regenerative properties.

In our study, the major protein that characterized the identified cell signature in TMS patients during relapse, is Arginase-1 (Arg-1). Arg-1 is a cytosolic enzyme that catalyzes the hydrolysis of L-arginine to urea and L-ornithine and is used to define the anti-inflammatory/alternative/M2 polarization state in macrophages. Arg-1 is expressed exclusively in infiltrating myeloid cells but not microglial cells in models of spinal cord injury and experimental autoimmune encephalomyelitis (EAE) (52). Previous studies showed arginase-1 to be strongly upregulated in the spinal cord of EAE mice, and mice treated with arginase inhibitors developed milder EAE with delayed onset, reduced disease score, and expedited recovery (53). Giles et al. (54) found that the Arg1+ CNS myeloid cells that accumulate in the CNS during EAE are derived, in part, from iNOS+ precursors. A recent study tried to track arginase-1 cells during neuroinflammation and found that Arginase-1/iNOS double-positive cells could represent a transition time-dependent event during the CNS inflammatory process. Most of these cells were predominantly found in the meninges and spinal cord, and it has been proposed that the local milieu and interaction between macrophages and CNS barrier cells can significantly shape the function of invading cells (55–57). Thus, further work is needed to delineate the nature, migration and phagocytosis properties of this myeloid subset as well as its role in T cell proliferation/activation.

Moreover, we found that the expanded myeloid cell population also expressed CD38, previously described as defining highly inflammatory macrophages and is robustly induced in human macrophages exposed to LPS ( ± IFN-γ) inflammatory stimuli (58, 59). Finally, regarding CD172 (SIRPa/b) expression on the identified myeloid population, it has been suggested that SIRPa on DCs is important for induction of the antigen-specific Th cells producing IL-17 and finally leading to the development of EAE (60, 61).

Pro-inflammatory monocytes are not only executors of inflammation but also may play an initiating role in various autoimmune diseases, and this has been nicely shown in animal models of MS, such as EAE (62, 63). The pathogenetic role of the recruitment of blood-derived myelomonocytic cells in the brain has been shown in animal models of MS like EAE, whereas it is unrelated to neurodegenerative conditions such as Huntington’s disease and amyotrophic lateral sclerosis (64). In mice, a recent very interesting study described five distinct peripheral blood monocyte populations present in different clinical stages of EAE, with varied frequencies. pSTAT3 upregulation occurred in peripheral monocytes during the active stages of EAE. Moreover, consistent with our results, peripheral monocyte populations have been shown to be multiple-cytokine-producing subsets in EAE (TNF-α+IL-6+ IL-10+). In contrast, multifunctional subsets in CNS-resident myeloid populations were only double positive, and these aberrancies were more prominent at the peak of the disease compared to the onset (63). CCR2-deficient mice that lack most circulating classical monocytes showed significant resistance to EAE induction (65), pointing to the pathogenic function of monocytes during disease development (2, 3, 63). One hypothesis regarding the pathogenesis of MS is that a soluble factor secreted by autoantigen reactive Th cells is implicated in the orchestration of inflammatory monocyte tissue infiltration. Towards this, mice deficient in CSF2, secreted in a RORγt-dependent manner by CD4+ T cells, are totally protected from EAE induction (66). CSF2R-signaling drives peripheral monocytes towards a highly inflammatory MHC-II+CD11c+ phenotype leading to CNS infiltration and tissue damage in an antigen-independent manner (4, 5). Another study that is in line with ours, applying CyTOF analysis in peripheral blood along with transcriptomics, showed that twins with MS shift in the myeloid compartment, away from non-classical monocytes, towards an inflammatory classical monocyte type. A subpopulation of monocytes exhibited elevated CCR2 and the GM-CSF receptor expression, indicative of sensitization towards inflammatory stimuli (67). Finally, we believe that TNF-a expression by our identified myeloid cell population is of pathogenetic relevance, as CNS-infiltrating macrophages have been shown to induce progressive EAE through sustained secretion of TNF (68).

The Janus face of myeloid cells in CNS immunity indicates that monocytes exacerbate tissue injury but also show remarkable growth-promoting and neuroprotective effects. Especially, immunomodulatory M2 Mϕ were essential for oligodendrocyte differentiation through activin-A production. Remyelination may require an initial early proinflammatory macrophage/microglia response (34, 69–72). In another study, the adoptive transfer of M2 microglia attenuated the severity of established MOG-EAE model in DBA/1 mice (73). Zhu et al. (74) described immunosuppressive Ly6Chi monocytes that expand in the periphery, accumulate in the CNS during EAE, and have the potential to differentiate into either iNOS+ or Arg1+ cells upon *ex vivo* culture with different polarizing factors. A question that arises is whether the phenotype of macrophages in the periphery can reflect the regenerative properties taking place inside the CNS at a specific time point. The TMS specific myeloid cell signatures we unraveled in this study could either promote disease or have regulatory functions. We cannot exclude the possibility that the expanded myeloid population found in TMS patients belongs to the monocytic-Myeloid-Derived Suppressor Cells (M-MDSCs, in humans, mainly expressing CD11b+CD14+CD15−CD33+HLA−DR−/low). MDSCs are immature myeloid cells that have emerged as a new cell type involved in the innate immune response, exerting a relevant suppressive effect over effector T cells in the context of MS (75–77). Interestingly, a higher density of M-MDSCs (Arginase-1 expressing) cells in demyelinated spinal cord lesions of EAE mice correlates with a higher density of Oligodendrocyte precursor cells or OPCs (NG2+ cells) in the adjacent periplaque, a function attributed to osteopontin secretion (78).

Pathological studies in PPMS have shown that the major myeloid cell type in the brain lesions is microglia, whereas only 20% are of macrophage origin (79, 80). M1-polarized macrophages/activated microglia at the lesion rim of slowly expanding lesions may serve as targets for new therapies in progressive MS (81). Regarding peripheral blood, in a recent study the analysis of monocyte subset revealed non-significantly elevated proportions of classical monocytes in RRMSi (inactive) patients and an increase in nonclassical monocytes in inactive PMS (PMSi) and active RRMS (RRMSa) participants (82). Herein we subgrouped PPMS patients into those with and without leptomeningeal enhancement based on MRI criteria. Leptomeningeal inflammation, given that it is more prevalent in the subset of patients with PPMS who had active disease and can be visualized on MRI (83, 84), may serve as a potential biomarker to identify patients with PPMS who may benefit most from B cell-directed therapy. Given the correlation between both ectopic lymphoid follicles (ELFs) in SPMS and widespread disorganized leptomeningeal inflammation in PPMS with adjacent cortical pathology, it is possible that leptomeningeal inflammation is an independent driver of disability, particularly in progressive MS (85–88). We found expansion of two populations of NK cells with a phenotype suggestive of NK with highly cytotoxic properties and one of them with features of terminally differentiated NK cells in PPMS patients with leptomeningeal enhancement (active PPMS), compared to those without (inactive PPMS). The role of NK cells in MS is rather controversial regarding knowledge coming from animal models of EAE with both protective/regulatory and deleterious effects (89, 90). A recent study found that NK cells migrate from the gut to the meninges and play a key role in regulating astrocyte function during EAE (91). Moreover, the involvement of CD56bright NK cells has been verified in MS periventricular pathology with CYTOF in postmortem tissue (92). Migration studies in an *in-vitro* model revealed that CD56bright NK possess a higher capacity barrier compared to their CD56dim counterparts (93).

Finally, we observed that selective depletion of B cells with anti- CD20 therapies, as well as glatiramer acetate reshapes the phenotype of myeloid cells. Importantly, the expansion of myeloid cells observed in one TMS patient during relapse was diminished after B cell depletion therapy, a notion that highlights disease-relevant crosstalk between these cell types. It has been shown that glatiramer acetate treatment decreases proinflammatory responses and antigen-presenting cell function in myeloid cells (94). Regarding anti-CD20 treatment, data are rather conflicting. GM-CSF+ B cells can contribute to proinflammatory macrophage responses *in vivo* in MS patients, and anti-CD20 diminishes macrophage proinflammatory responses in these treated patients due to the removal of GM-CSF+ B cells (95). Nevertheless, another study showed that anti-CD20 treatment increased the relative frequency of monocytes and accentuated pro-inflammatory monocyte function (96). One reason for this discrepancy could be the different disease stages of MS patients during sample collection.

### 4.1 Limitation

We cannot eliminate the confounding effects of therapeutic heterogeneity, while the relatively small number of cases per group, especially when also accounting for disease stage, could pose another limitation. The combined contribution of multifaceted risk factors for the onset of multiple sclerosis such as genetic and environmental factors may have affected our results. Moreover, analysis of matched samples with CSF could better illustrate the ensuing pathogenetic mechanisms. Despite the apparent differences that we found in the MS and glatiramer acetate or anti-CD20-MS cohorts, we cannot conclude these effects are caused by glatiramer acetate or anti-CD20 treatment, and future studies assessing monocyte phenotype and responses before and after initiation of treatment in the same MS population will help to establish causality. Nevertheless, as a proof-of-concept study, it demonstrates the ability of single-cell mass cytometry to reveal the heterogeneity of the myeloid cell population and to differentiate MS clinical subtypes and disease stages (relapse, remission).

Based on our results, we cannot conclude if there is a pathogenetic link between arginase-expressing macrophages and lymphopenia seen in CD4+ T cells and specifically in Th2-like cells in TMS patients during relapse. However, we hypothesize that Arg1-expressing macrophages could alter the balance among Th1/Th2-dependent inflammation, as Arg1-expressing macrophages suppress Th2 responses and lead to decreases in CD4+ T cell responses (97, 98). On the other hand, CD4+ T cell lymphopenia could represent a risk factor for CNS autoimmunity, as shown in Lewis rats with T cell lymphopenia and aggravated EAE. Regardless of underlying pathology, chronic lymphopenia has been associated with an increased incidence of autoimmunity (99–102).

## 5 Conclusion

In conclusion, we identified for the first time an enrichment of myeloid-specific signatures in TMS patients during disease relapse, at early disease settings, distinguishing them from all disease subgroups; this specific myeloid cell expansion appeared amenable to therapeutic manipulations with glatiramer acetate and anti-CD20 monoclonal antibody. Our analysis separating PPMS patients based on leptomeningeal enhancement provides initial evidence of aberrant NK cell activation profile (highly cytolytic and terminally differentiated NK cells) specifically in those with disease activity, paving thus the way for future targeted therapies in this disease group, that represents an unmet medical need. Radiological activity in PPMS is challenging to capture with classic ways (gadolinium enhancement), as most lesions are silent. So, findings from this study open the way for discovering research tools for easily accessible markers such as MRI-based and peripheral blood cell signatures for monitoring disease evolution in the era of new drug discovery.

Previous studies in the field have not revealed major immunological differences between patients with progressive forms of MS and healthy volunteers. There is an absolute need for emerging biomarkers in tracking the activity of specific immune cell subsets to guide more targeted therapies in MS. We believe that the identified myeloid-specific signature of Arginase-1^+^, TNFa^+^ cells in TMS patients during their relapse provides a diagnostic biomarker easily accessible in the peripheral blood. It is currently unknown which are the functional properties of the identified cell population in TMS patients. Future studies will focus on the MS specific microenvironment able to polarize macrophages in this unique cell signature and will aim to assess its role in either MS disease perpetuation or resolution. The functional properties of such a population could pave the way for novel therapeutic approaches that specifically target and inhibit peripheral monocyte trafficking into the CNS. This strategy might be more relevant for specific MS disease forms like TMS and may even have potentially fewer deleterious side effects than existing MS therapies.

## Data availability statement

The data presented in the study are deposited in the Flow Repository with accession number FR-FCN-Z5NY (http://flowrepository.org/id/FR-FCM-Z5NY).

## Ethics statement

The studies involving human participants were reviewed and approved by Ethics Committee of Aeginition University Hospital with number 689/07.10.21. The patients/participants provided their written informed consent to participate in this study.

## Author contributions

AV, NP, CK and LS: study conception and design. AV and NP: literature search. M-EE, CK, LS: recruited and treated patients. XT: sample collection and processing, AV, NP, CG: acquisition of data. NP, AV, EP, DK, CS-N: data analysis and interpretation, EP: bioinformatic analysis, GV: MRI acquisition, analysis, and interpretation. All authors contributed to the article and approved the submitted version.

## Supplementary material

Supplementary Figure 1 Validation and titration of in-house conjugated antibodies. **(A)** Validation and titration of anti-CD192 conjugated in-house with Cadmium 109 (109Cd). Different dilutions of the stock antibody prepared (1:100, 1:200, 1:400 and 1:800) were used to stain PBMCs as described in the Materials and Methods section. Acquired data (fcs files) were normalized and uploaded to Cytobank. Following data cleanup, gated CD192- and CD192+ populations were used to calculate median and 95% values for separation index (SI) calculation. **(B)** Data files from different dilutions were also concatenated and analyzed in FlowJo to visually inspect overall staining quality. Circle and box in red represent the selected dilution used throughout the study. This analysis is a representative example that was used for all other in-house conjugated antibodies used in this study.

Supplementary Figure 2 Data cleanup strategy and quality assessment. **(A)** Acquired data were normalized and fcs files were loaded into FlowJo software for bivariate cleanup workflow developed by Standard Biotools Inc. (formerly Fluidigm) and Verity Software House. This workflow included removal of cellular aggregates and ion cloud fusions using gating for ^140^Ce, ^191/193^Ir and gaussian parameters center, width, offset and residual. **(B)** Quality check on the data for bead-cell aggregates. Workflow in this analysis is a representative example that was used for all the fcs files generated in this study.

Supplementary Figure 3 Batch effect control. The batch effect has been controlled on the overview level (t-SNE maps) for **(A)** different acquisition days as well as **(B)** different antibody mixes used.

Supplementary Figure 4 Radiological characteristics of two patients with tumefactive lesions at disease exacerbation and during remission. **(A–F)** Patient 1 presented with two simultaneous TDLs at initial attack, located in the middle cerebellar peduncles **(A)** and in the parietal lobe **(C)**. The two lesions showed gadolinium enhancement with open ring enhancement pattern **(B, D)**. Arrows indicate the enhancement patterns. Another patient (Patient-2) during clinical attack presented with one TDL lesion (more than 5cm in length), with heterogenous gadolinium enhancement, as indicated with arrow **(E, F)**. Patient 3, with a known biopsied Marburg-like TMS diagnosed 5 years before, was in disease remission during sample collection. MRI scanning is provided during remission with no MRI activity **(H)**. A fourth patient with known MS during a severe relapse presented with a TDL lesion with closed ring-like enhancement pattern **(I, J)**. A fifth patient displayed a TDL lesion with a patchy contrast enhancement pattern. T2-weighted image: **(A, G)**. FLAIR images: **(C, E, I, K)**, T1-weighted contrast-enhanced images: **(B, D, F, H, J, L)**. FLAIR, fluid-attenuated inversion recovery; TDL, tumefactive demyelinating lesion, SWI, susceptibility weighted imaging; TMS, Tumefactive Multiple Sclerosis; MRI, magnetic resonance imaging.

Supplementary Figure 5 Detection of leptomeningeal contrast enhancement in two PPMS patients using 3 Tesla MRI. **(A–C)** MRI images of Patient-1 with one representative nodular foci indicative of leptomeningeal enhancement in the temporal lobe. **(D–F)** MRI images of Patient-2 with one representative linear foci indicative of leptomeningeal enhancement in the parietal lobe. Arrows on 3D-FLAIRGd images **(A, D)** indicate foci of hyper-intensities, without corresponding hyper-intensities on 3D-FLAIR **(B, E)** and 3D-T1wGd **(C, F)** images, representing LMCE. PPMS, Primary Progressive Multiple Sclerosis; MRI, magnetic resonance imaging; 3D-FLAIR, three-dimensional fluid-attenuated inversion recovery; 3D-FLAIRGd, 3D-FLAIR post-gadolinium; 3D-T1wGd, three-dimensional -T1-weighted post- gadolinium; LMCE, leptomeningeal contrast enhancement.

Supplementary Figure 6 Peripheral blood cell counts by whole blood routine analysis in 3 groups of MS patients. Box plots are showing frequency of each immune cell subset in peripheral blood of MS patients (PPMS; n=8, RRMS; n=8, TMS; n=10). Only drug-naïve patients are included in the analysis. Cells are expressed as % positive or as absolute numbers. p < 0.05 was considered significant, non-parametric Kruskal Wallis test with correction for multiple comparisons (Dunn’s test) was applied. WBC, white blood cells; NEU, neutrophils; LYM, lymphocytes; MONO, monocytes; PPMS, Primary Progressive Multiple Sclerosis; RRMS, Relapsing Remitting Multiple Sclerosis; TMS, Tumefactive Multiple Sclerosis.

Supplementary Figure 7 Representative staining patterns of 44 markers displayed in t-SNE CUDA algorithm. t-SNE maps of all markers included in the analysis. A representative example from one patient of the cohort is shown here as an example. Each dot in the map represents a single cell and color indicates the intensity of each marker on display (red for high and blue for low expression). Gates in these maps were generated, and automatically applied to all maps, manually in Cytobank gating editor, based on the expression of key lineage markers such as CD3, CD4, CD8, CD19, CD11c, TCRgd, CD56 and CD123 to identify major “islands” of immune subsets for subsequent deeper analysis (*shown in main* ).

Supplementary Figure 8 Differences in major immune cell subsets among MS disease subgroups and healthy controls. Box plots showing frequency (expressed as % of live singlet CD45+ cells) of each major immune cell subset in peripheral blood mononuclear cells of MS patients and healthy donors. p < 0.05 was considered significant, non-parametric Kruskal Wallis test with correction for multiple comparisons (FDR) was applied. T4; CD4+ T cells, T8; CD8+ T cells, M, Myeloid cells; D, Dendritic cells; NK, natural killer cells.

Supplementary Figure 9 Deep phenotypic analysis of peripheral blood immune subsets with FlowSOM clustering. FlowSOM generated metaclusters (n=50) projected on the tSNE map for visualization. The heatmap includes metacluster numbers and expression of major lineage markers with which we grouped metaclusters belonging to the major immune cell lineages (CD3, CD4, CD8, TCRgd, CD19, CD56, CD11c, CD123). Cluster frequencies (of total CD45+ cells) are also shown with the bar graph on the right. T4; CD4+ T cells, T8; CD8+ T cells, M, Myeloid cells; D, Dendritic cells; NK, natural killer cells.

Supplementary Figure 10 Differences in major immune cell subsets among MS disease subgroups and healthy controls. **(A**–**E)** Box plots showing the frequency (expressed as % of CD45+ cells) of each FlowSOM generated metacluster in peripheral blood mononuclear cells of MS patients (n=27; PPMS=8, RRMS=9, TMS=10) and healthy donors. (n=10). The number below each plot represents the generated metacluster. PPMS, Primary Progressive Multiple Sclerosis; RRMS, Relapsing Remitting Multiple Sclerosis; TMS, Tumefactive Multiple Sclerosis; HD, Healthy donors.

Supplementary Figure 11 Validation of myeloid cell expansion in TMS patients with flow cytometry. **(A)** Gating strategy for the myeloid immune cell phenotype (same cohort that one used in CyTOF). Representative paradigm from one patient is shown in A and generated with FlowJo program. Myeloid cells defined as CD3/CD19/CD56 negative cells (total myeloid) were separated in two groups based on their forward side scattered properties: in those with higher FSC features (FSC high Myeloid) and those with lower FSC features. FSC high Myeloid cell population co-expressed the higher levels of CD11c and CD14, compared to FSC low cells, possibly indicative of their activation status. **(B, C)** Representative flow cytometry plots from each patient from the 4 groups of patients and one healthy control are presented according to gating strategies shown in A. **(D)** Comparative assessment of the frequency of each myeloid cell population among healthy controls (n=5), RRMS patients in remission (n=4), RRMS patients in relapse (n=4), TMS patients in remission (n=5) and TMS patients in relapse (n=5). RRMS, Relapsing Remitting Multiple Sclerosis; Rem, Remission; Rel, Relapse; TMS, Tumefactive Multiple Sclerosis; HD, Healthy donors; FSC, forward side scatter; PBMC, peripheral blood mononuclear cells.

Supplementary Figure 12 Increased serum neurofilament light chains reflect disease activity in TMS patients and correlate with the expansion of the myeloid cell signature. **(A)** Serum neurofilament light chains were measured in duplicates using the high-sensitivity single-molecule array (Simoa) NF-Light Advantage Kit (Quanterix), according to the manufacturer’s instructions on an HDX platform (Quanterix). **(B)** Serum NfL in RRMS and TMS patients. **(C)** Correlation among NfL and myeloid cell signatures as defined by analysis of CytOF (also see ) and conventional flow cytometry data analysis. RRMS, Relapsing-Remitting Multiple Sclerosis; TMS, Tumefactive Multiple Sclerosis; Macro, macrophages; Rem, Remission; Rel, Relapse; NfL, neurofilament light chains; SD, standard deviation.

Supplementary Figure 13 Differences in CD8+ T cells among MS disease subgroups and healthy controls. **(A)** FlowSOM generated metaclusters for the CD8+ T cell lineage projected on the tSNE map. **(B)** Heatmap showing, for each of the clusters generated, the average intensity of each lineage marker across all cells. A tentative biological name was assigned to each metacluster based on the most abundant CD8+ T cell related marker expressed by each cluster. **(C)** MS patients stratified in different disease subgroups (PPMS; n=8, RRMS; n=9, TMS; n=10). RRMS and TMS were further subdivided in those in remission (RRMS; n=4, TMS; n=5) and relapse (RRMS; n=5, TMS; n=5). Box plots showing the frequency (expressed as % of CD45+ cells) of each major immune cell subset in peripheral blood mononuclear cells of patient subgroup and healthy donors (n=10). Each dot represents the value of each sample. p < 0.05 was considered significant, non-parametric Kruskal Wallis test with correction for multiple comparisons (FDR) was applied. PPMS, Primary Progressive Multiple Sclerosis; RRMS, Relapsing Remitting Multiple Sclerosis; Rem, Remission; Rel, Relapse; HD, Healthy donors; TMS, Tumefactive Multiple Sclerosis; FlowSOM, Flow Self-Organizing Map; FDR, false discovery rate.

Supplementary Figure 14 Higher abundance of a memory double negative B cell subset in TMS patients during relapse. **(A)** FlowSOM generated metaclusters for the B cell lineage projected on the tSNE map. **(B)** Heatmap showing, for each of the metaclusters generated, the average intensity of each B cell related marker (stage or activation marker). A tentative biological name was assigned to each metacluster based on the most abundant B cell related marker expressed by each cluster. **(C)** MS patients stratified in different disease subgroups (PPMS; n=9, RRMS; n=9, TMS; n=10). RRMS and TMS were further subdivided in those in remission (RRMS; n=4, TMS; n=5) and relapse (RRMS; n=5, TMS; n=5). Box-plots show frequencies (%) of the indicative cell types. Each dot represents the value of each sample. p < 0.05 was considered significant, non-parametric Kruskal Wallis test with correction for multiple comparisons (FDR) was applied. PPMS, Primary Progressive Multiple Sclerosis; RRMS, Relapsing Remitting Multiple Sclerosis; Rem, Remission; Rel, Relapse; TMS, Tumefactive Multiple Sclerosis; HD, Healthy donors, t-SNE, t-distributed stochastic neighbor embedding; FlowSOM, Flow Self-Organizing Map; PD-L1, Programmed death-ligand 1; TNF-α, tumor necrosis factor alpha; IL-10, Interleukin 10; IL-6, Interleukin 6; CCR, CC chemokine receptors; CXCR, CXC chemokine receptor; FDR, false discovery rate.

Supplementary Figure 15 tSNE plots of one TMS patient during relapse and after anti-CD20 treatment. TMS, Tumefactive Multiple Sclerosis; CCR2 or CD192, C-C chemokine receptor type 2; TNF-α, tumor necrosis factor alpha; IL-6, Interleukin 6.

Supplementary Figure 16 Schematic representation of cell-types differentially expressed in peripheral blood as revealed by broad immunophenotyping with CyTOF analysis. The cross-talks among cell types are based on statistically significant correlations as revealed by correlation matrix among all of immune subsets in relapsing MS (RRMS and TMS) patients as shown in . The image is hypothesis driven and implicates different cell drivers in different clinical subtypes of MS patients. PPMS, Primary Progressive Multiple Sclerosis; RRMS, Relapsing Remitting Multiple Sclerosis; TMS, Tumefactive Multiple Sclerosis.
