# Supplementary figures and images for: Specific myeloid signatures in peripheral blood differentiate active and rare clinical phenotypes of multiple sclerosis
Source: Front Immunol. 2023 Jan 25;14:1071623. doi: 10.3389/fimmu.2023.1071623 (PMC9905713; doi:10.3389/fimmu.2023.1071623)

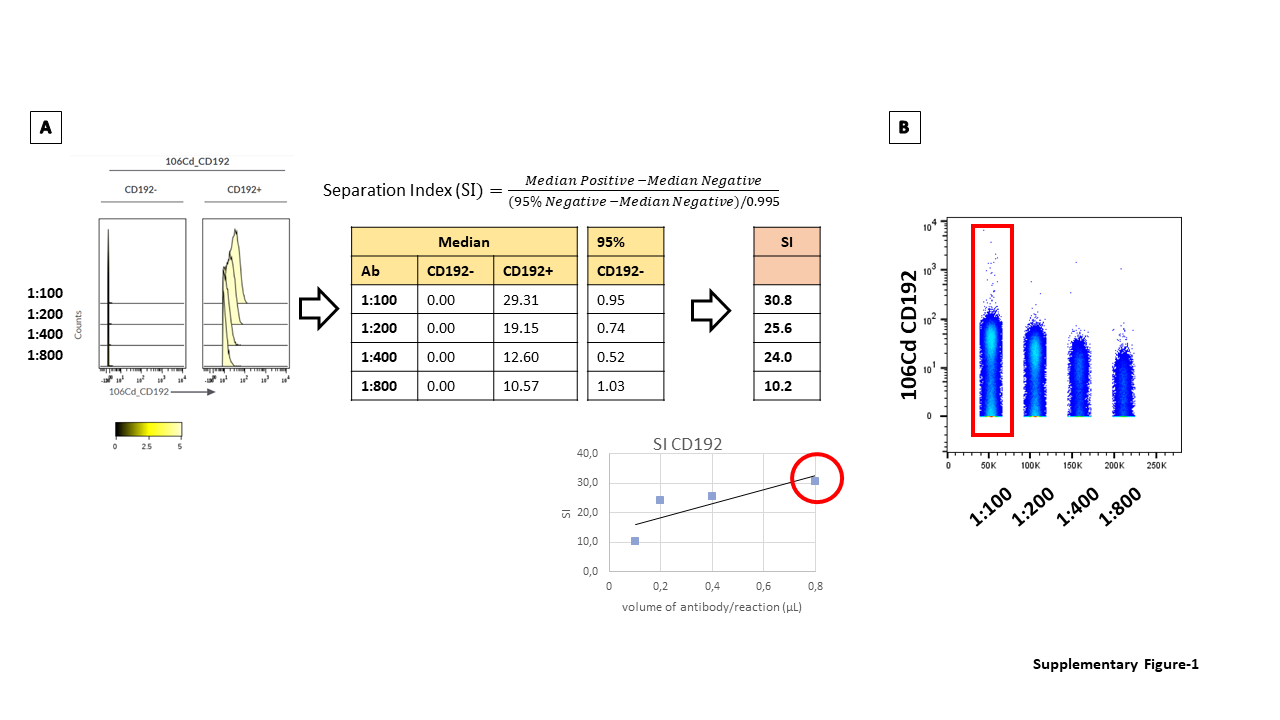

Supplement: Supplementary Figure 1 — Validation and titration of in-house conjugated antibodies. (A) Validation and titration of anti-CD192 conjugated in-house with Cadmium 109 (109Cd). Different dilutions of the stock antibody prepared (1:100, 1:200, 1:400 and 1:800) were used to stain PBMCs as described in the Materials and Methods section. Acquired data (fcs files) were normalized and uploaded to Cytobank. Following data cleanup, gated CD192- and CD192+ populations were used to calculate median and 95% values for separation index (SI) calculation. (B) Data files from different dilutions were also concatenated and analyzed in FlowJo to visually inspect overall staining quality. Circle and box in red represent the selected dilution used throughout the study. This analysis is a representative example that was used for all other in-house conjugated antibodies used in this study. [file Image_1.tif]

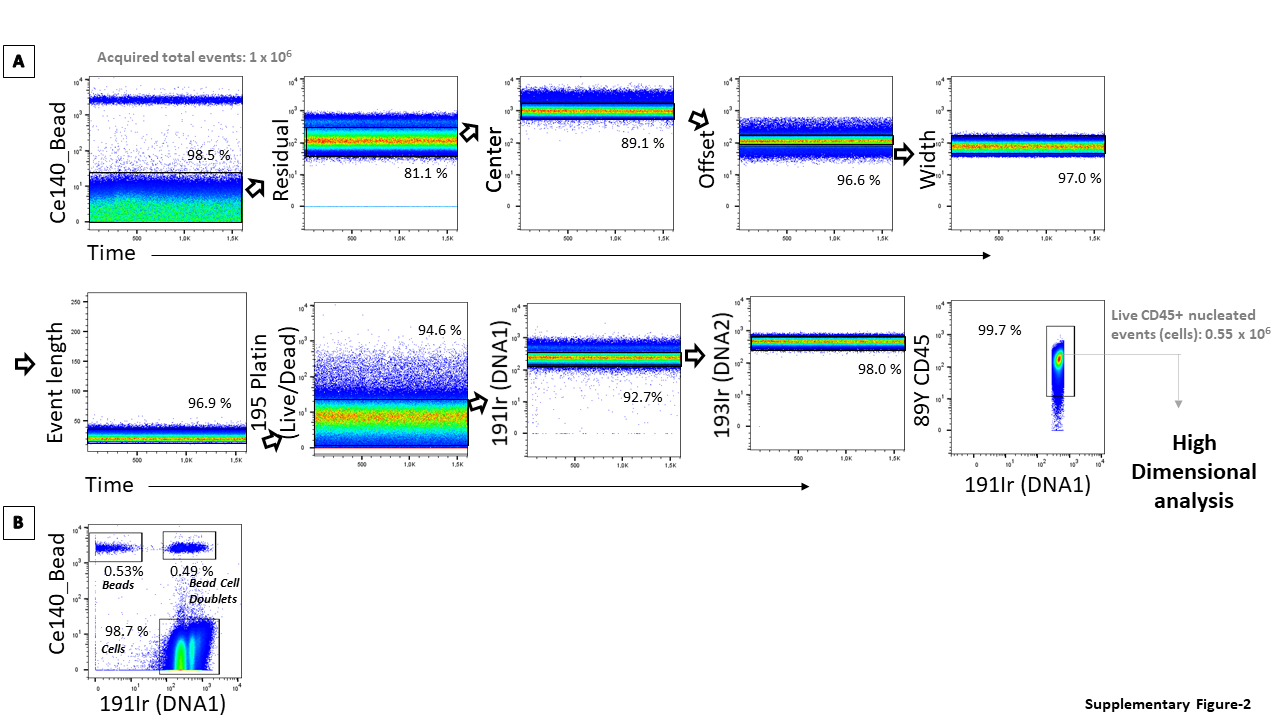

Supplement: Supplementary Figure 2 — Data cleanup strategy and quality assessment. (A) Acquired data were normalized and fcs files were loaded into FlowJo software for bivariate cleanup workflow developed by Standard Biotools Inc. (formerly Fluidigm) and Verity Software House. This workflow included removal of cellular aggregates and ion cloud fusions using gating for 140Ce, 191/193Ir and gaussian parameters center, width, offset and residual. (B) Quality check on the data for bead-cell aggregates. Workflow in this analysis is a representative example that was used for all the fcs files generated in this study. [file Image_2.tif]

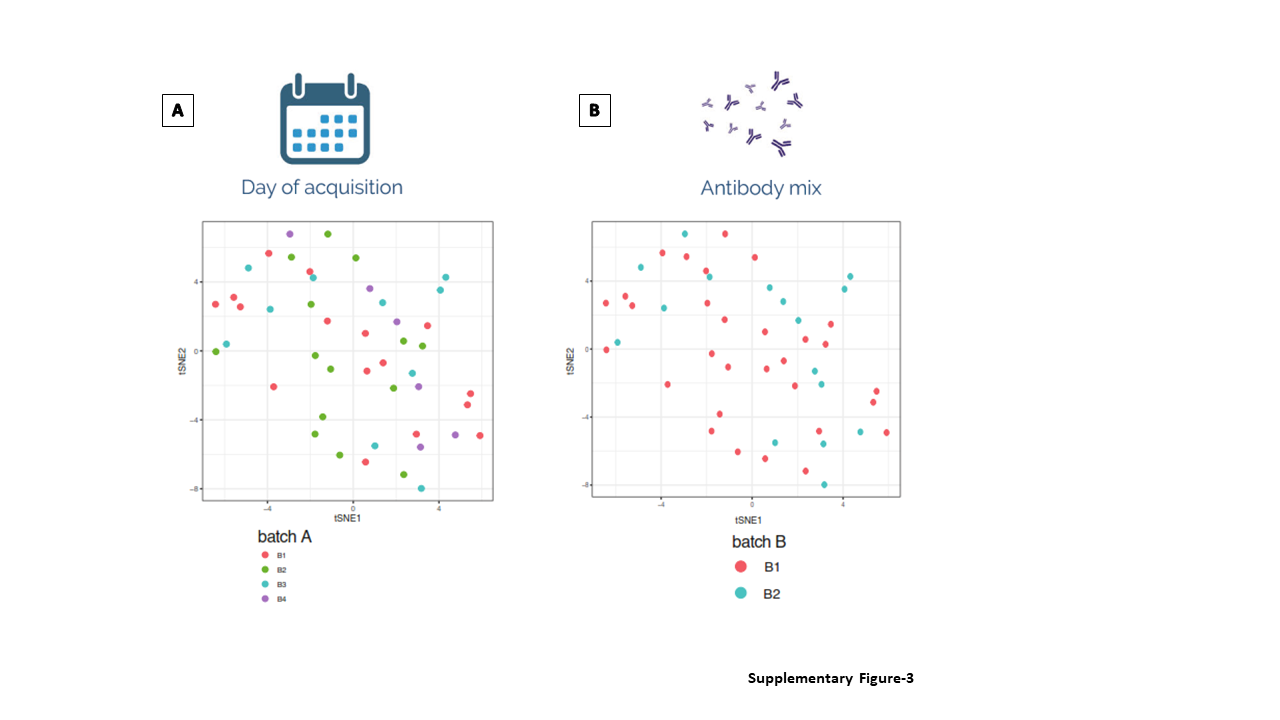

Supplement: Supplementary Figure 3 — Batch effect control. The batch effect has been controlled on the overview level (t-SNE maps) for (A) different acquisition days as well as (B) different antibody mixes used. [file Image_3.tif]

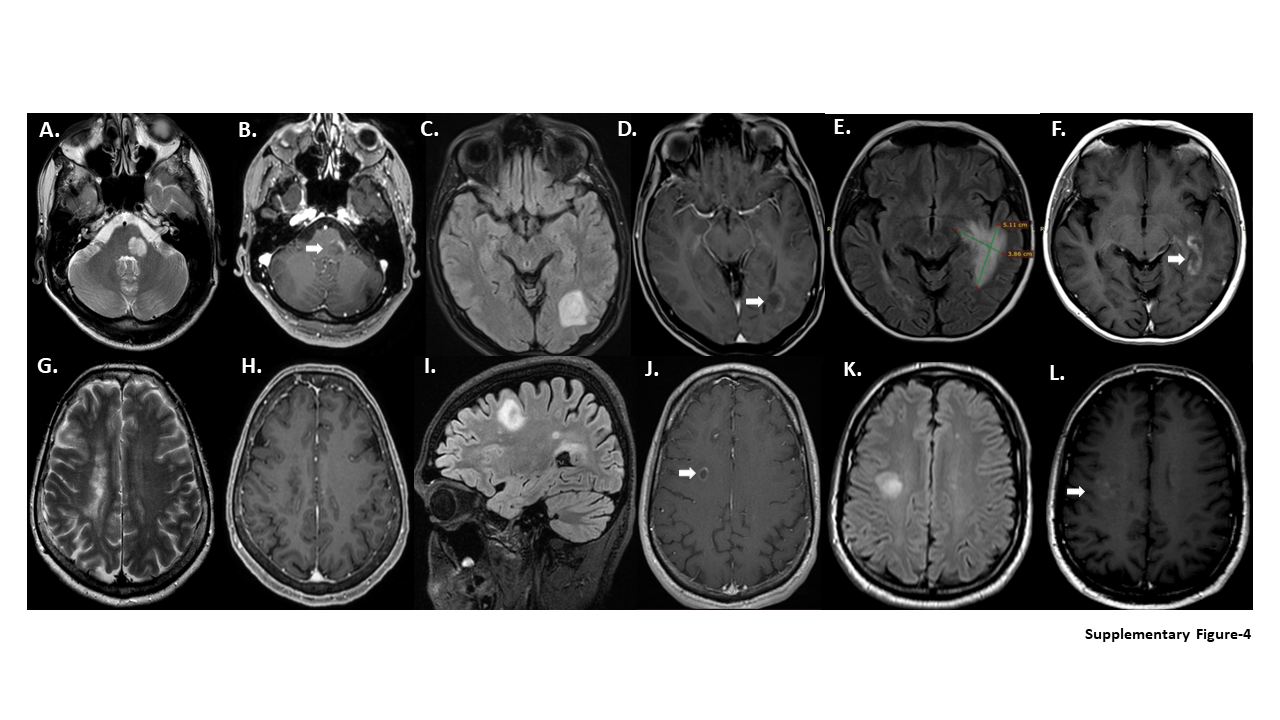

Supplement: Supplementary Figure 4 — Radiological characteristics of two patients with tumefactive lesions at disease exacerbation and during remission. (A–F) Patient 1 presented with two simultaneous TDLs at initial attack, located in the middle cerebellar peduncles (A) and in the parietal lobe (C). The two lesions showed gadolinium enhancement with open ring enhancement pattern (B, D). Arrows indicate the enhancement patterns. Another patient (Patient-2) during clinical attack presented with one TDL lesion (more than 5cm in length), with heterogenous gadolinium enhancement, as indicated with arrow (E, F). Patient 3, with a known biopsied Marburg-like TMS diagnosed 5 years before, was in disease remission during sample collection. MRI scanning is provided during remission with no MRI activity (H). A fourth patient with known MS during a severe relapse presented with a TDL lesion with closed ring-like enhancement pattern (I, J). A fifth patient displayed a TDL lesion with a patchy contrast enhancement pattern. T2-weighted image: (A, G). FLAIR images: (C, E, I, K), T1-weighted contrast-enhanced images: (B, D, F, H, J, L). FLAIR, fluid-attenuated inversion recovery; TDL, tumefactive demyelinating lesion, SWI, susceptibility weighted imaging; TMS, Tumefactive Multiple Sclerosis; MRI, magnetic resonance imaging. [file Image_4.tif]

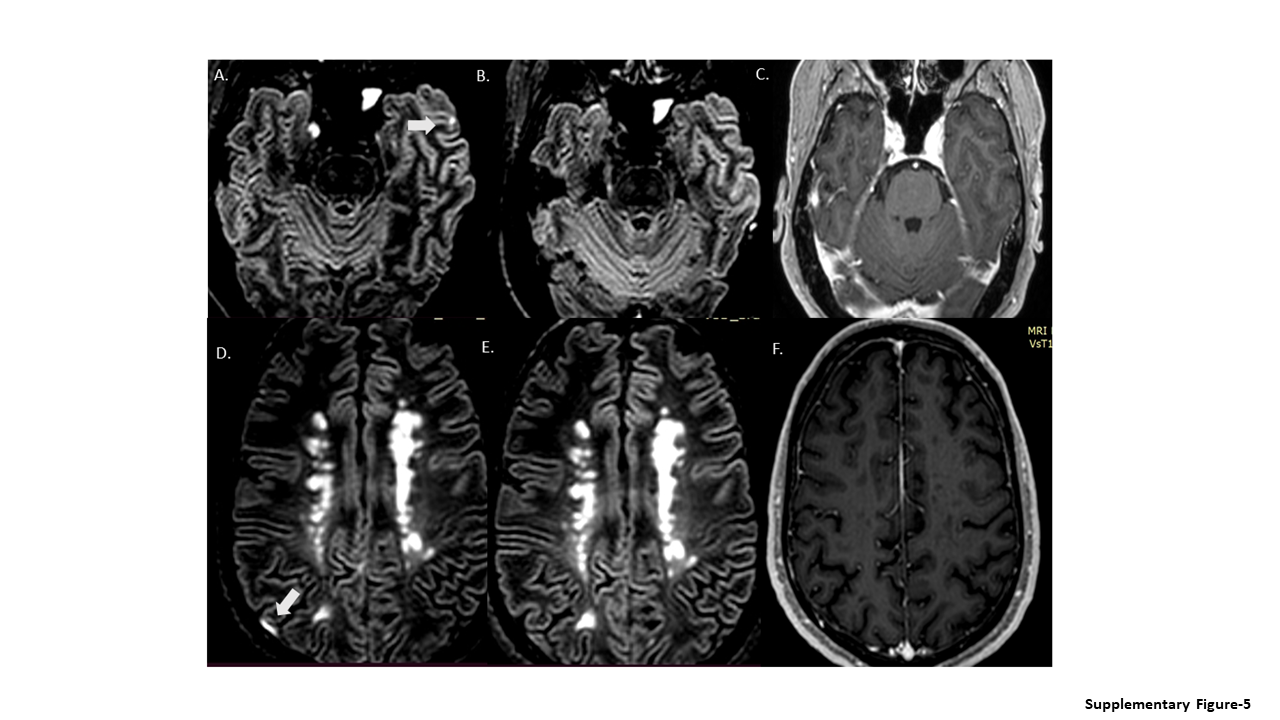

Supplement: Supplementary Figure 5 — Detection of leptomeningeal contrast enhancement in two PPMS patients using 3 Tesla MRI. (A–C) MRI images of Patient-1 with one representative nodular foci indicative of leptomeningeal enhancement in the temporal lobe. (D–F) MRI images of Patient-2 with one representative linear foci indicative of leptomeningeal enhancement in the parietal lobe. Arrows on 3D-FLAIRGd images (A, D) indicate foci of hyper-intensities, without corresponding hyper-intensities on 3D-FLAIR (B, E) and 3D-T1wGd (C, F) images, representing LMCE. PPMS, Primary Progressive Multiple Sclerosis; MRI, magnetic resonance imaging; 3D-FLAIR, three-dimensional fluid-attenuated inversion recovery; 3D-FLAIRGd, 3D-FLAIR post-gadolinium; 3D-T1wGd, three-dimensional -T1-weighted post- gadolinium; LMCE, leptomeningeal contrast enhancement. [file Image_5.tif]

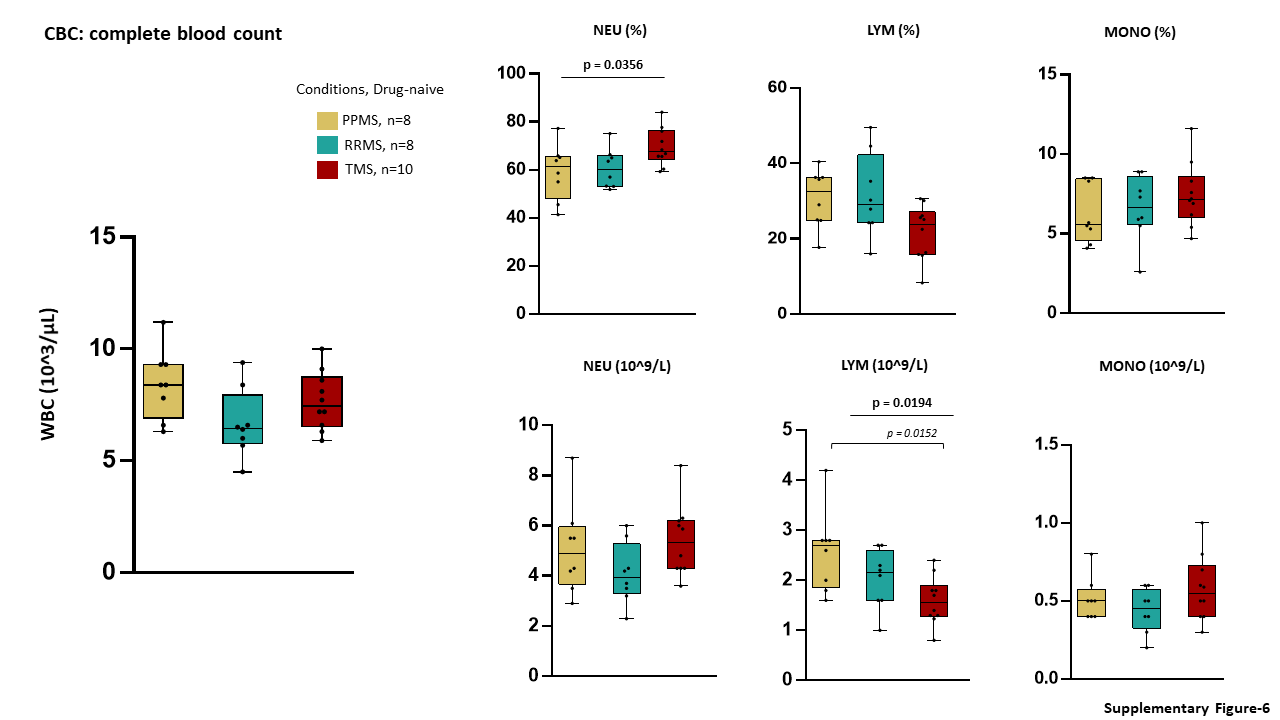

Supplement: Supplementary Figure 6 — Peripheral blood cell counts by whole blood routine analysis in 3 groups of MS patients. Box plots are showing frequency of each immune cell subset in peripheral blood of MS patients (PPMS; n=8, RRMS; n=8, TMS; n=10). Only drug-naïve patients are included in the analysis. Cells are expressed as % positive or as absolute numbers. p < 0.05 was considered significant, non-parametric Kruskal Wallis test with correction for multiple comparisons (Dunn’s test) was applied. WBC, white blood cells; NEU, neutrophils; LYM, lymphocytes; MONO, monocytes; PPMS, Primary Progressive Multiple Sclerosis; RRMS, Relapsing Remitting Multiple Sclerosis; TMS, Tumefactive Multiple Sclerosis. [file Image_6.tif]

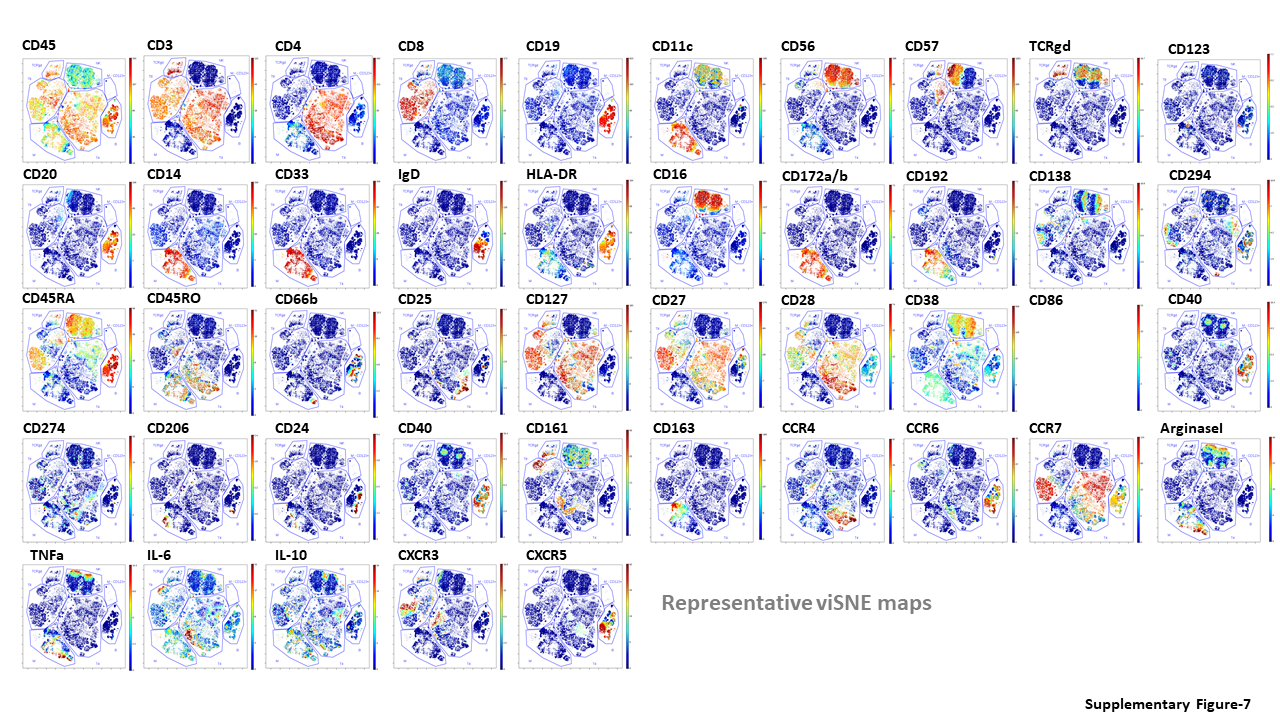

Supplement: Supplementary Figure 7 — Representative staining patterns of 44 markers displayed in t-SNE CUDA algorithm. t-SNE maps of all markers included in the analysis. A representative example from one patient of the cohort is shown here as an example. Each dot in the map represents a single cell and color indicates the intensity of each marker on display (red for high and blue for low expression). Gates in these maps were generated, and automatically applied to all maps, manually in Cytobank gating editor, based on the expression of key lineage markers such as CD3, CD4, CD8, CD19, CD11c, TCRgd, CD56 and CD123 to identify major “islands” of immune subsets for subsequent deeper analysis (shown in main ). [file Image_7.tif]

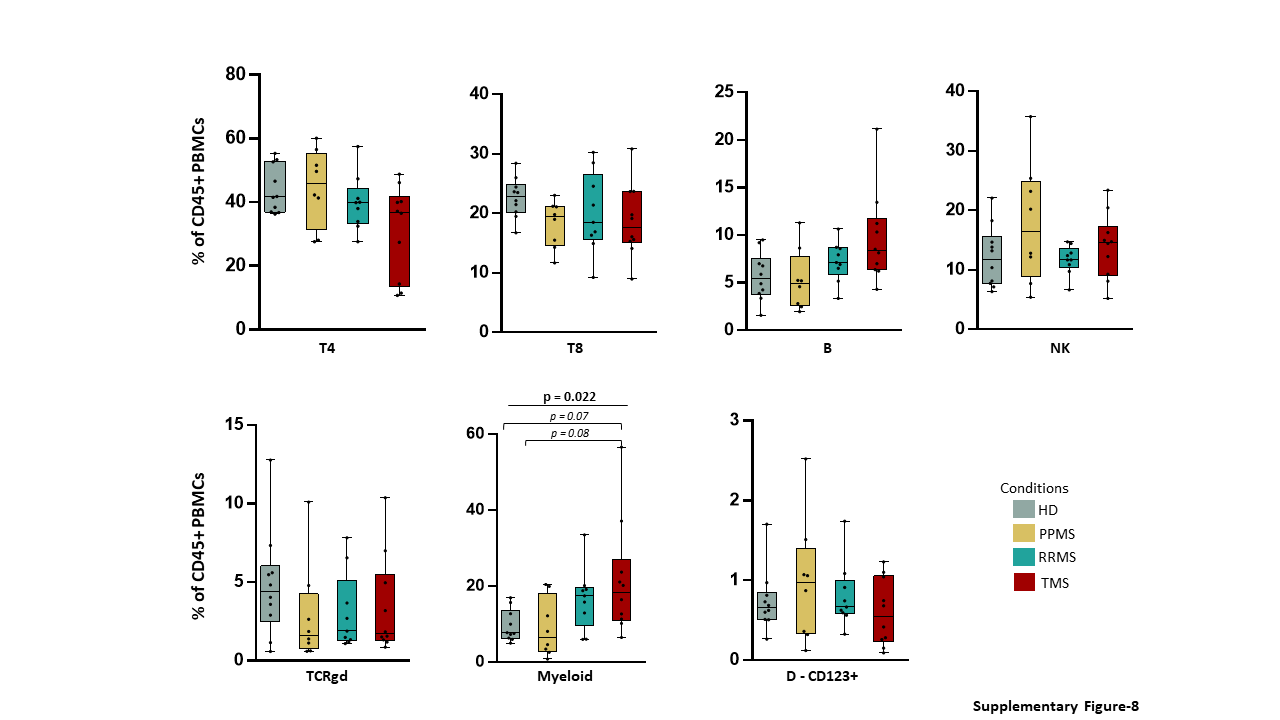

Supplement: Supplementary Figure 8 — Differences in major immune cell subsets among MS disease subgroups and healthy controls. Box plots showing frequency (expressed as % of live singlet CD45+ cells) of each major immune cell subset in peripheral blood mononuclear cells of MS patients and healthy donors. p < 0.05 was considered significant, non-parametric Kruskal Wallis test with correction for multiple comparisons (FDR) was applied. T4; CD4+ T cells, T8; CD8+ T cells, M, Myeloid cells; D, Dendritic cells; NK, natural killer cells. [file Image_8.tif]

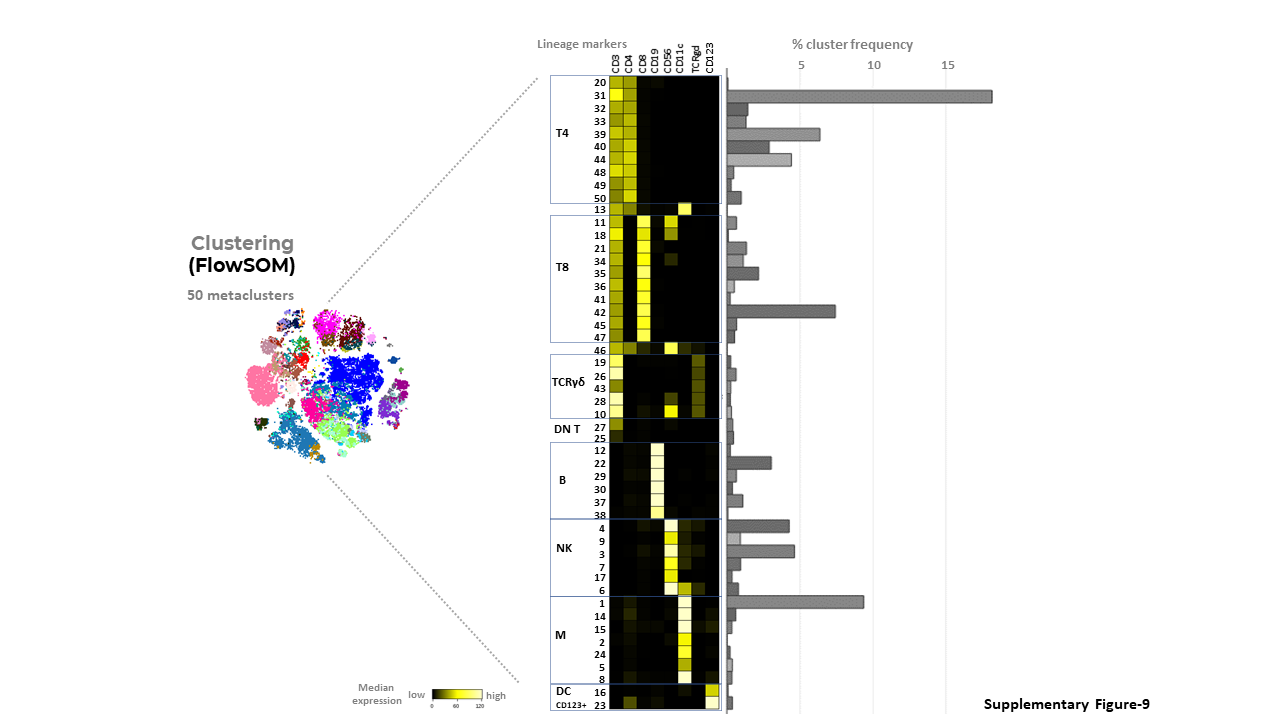

Supplement: Supplementary Figure 9 — Deep phenotypic analysis of peripheral blood immune subsets with FlowSOM clustering. FlowSOM generated metaclusters (n=50) projected on the tSNE map for visualization. The heatmap includes metacluster numbers and expression of major lineage markers with which we grouped metaclusters belonging to the major immune cell lineages (CD3, CD4, CD8, TCRgd, CD19, CD56, CD11c, CD123). Cluster frequencies (of total CD45+ cells) are also shown with the bar graph on the right. T4; CD4+ T cells, T8; CD8+ T cells, M, Myeloid cells; D, Dendritic cells; NK, natural killer cells. [file Image_9.tif]

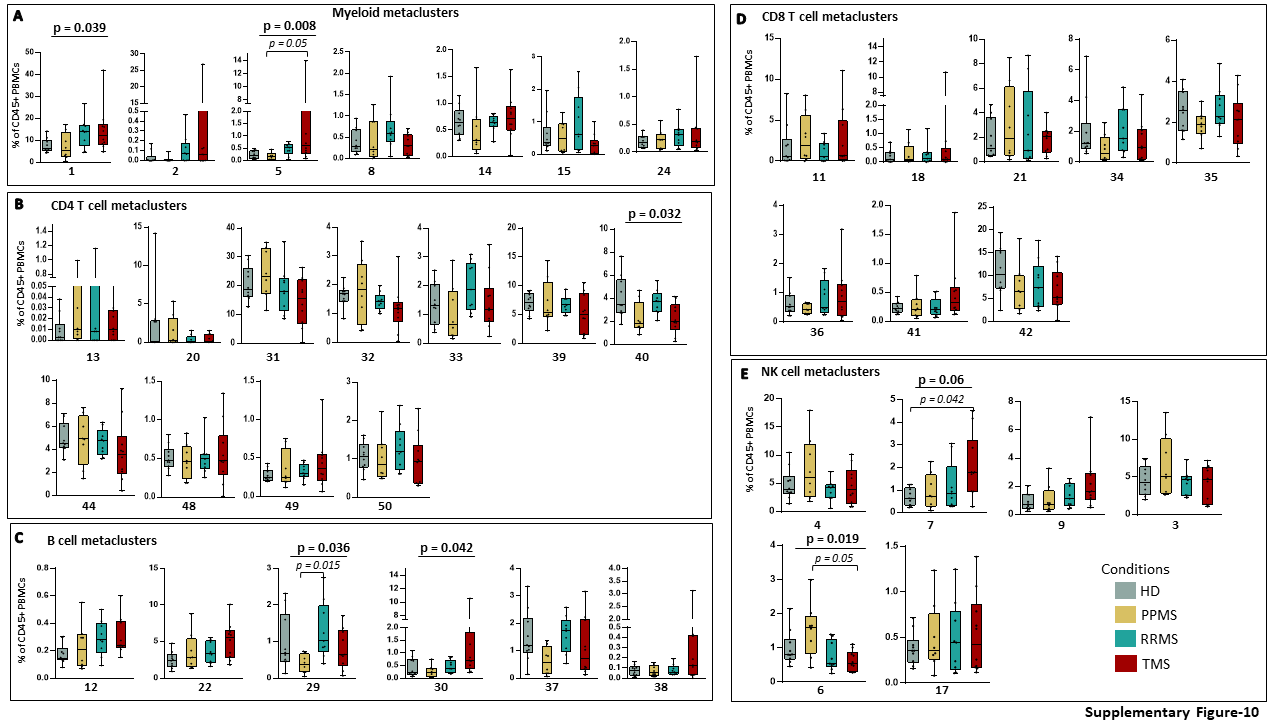

Supplement: Supplementary Figure 10 — Differences in major immune cell subsets among MS disease subgroups and healthy controls. (A–E) Box plots showing the frequency (expressed as % of CD45+ cells) of each FlowSOM generated metacluster in peripheral blood mononuclear cells of MS patients (n=27; PPMS=8, RRMS=9, TMS=10) and healthy donors. (n=10). The number below each plot represents the generated metacluster. PPMS, Primary Progressive Multiple Sclerosis; RRMS, Relapsing Remitting Multiple Sclerosis; TMS, Tumefactive Multiple Sclerosis; HD, Healthy donors. [file Image_10.tif]

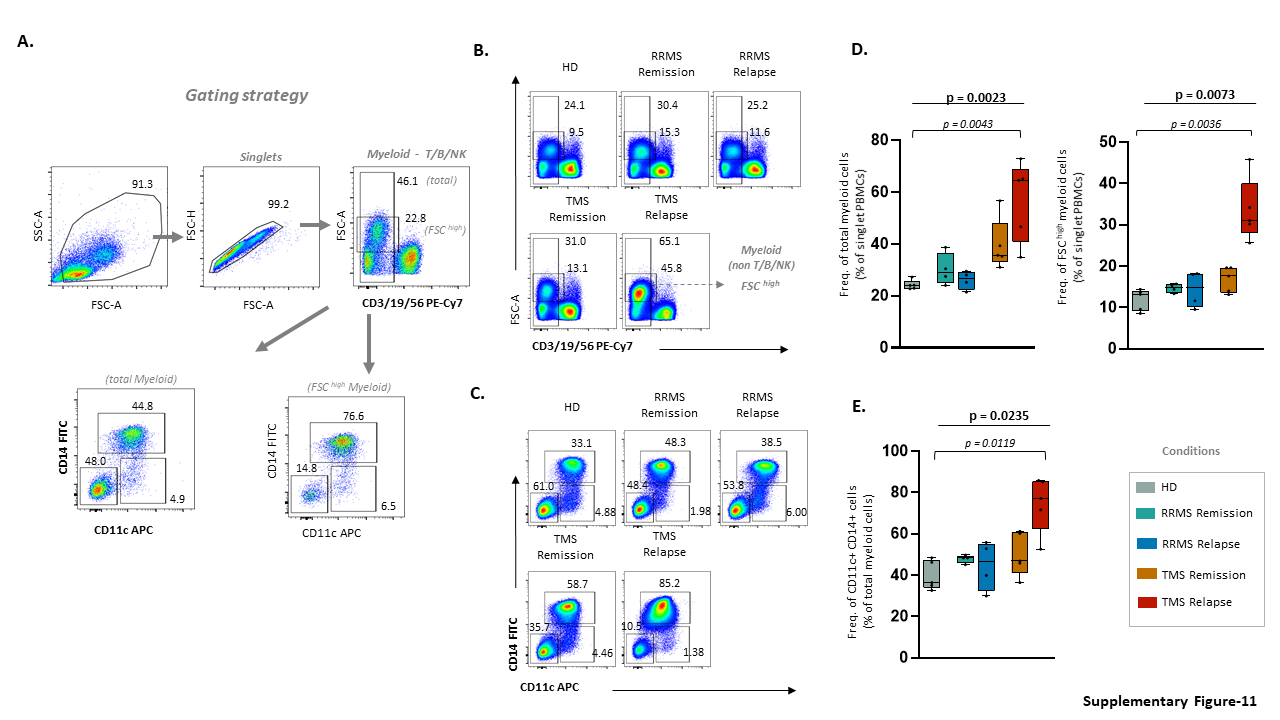

Supplement: Supplementary Figure 11 — Validation of myeloid cell expansion in TMS patients with flow cytometry. (A) Gating strategy for the myeloid immune cell phenotype (same cohort that one used in CyTOF). Representative paradigm from one patient is shown in A and generated with FlowJo program. Myeloid cells defined as CD3/CD19/CD56 negative cells (total myeloid) were separated in two groups based on their forward side scattered properties: in those with higher FSC features (FSC high Myeloid) and those with lower FSC features. FSC high Myeloid cell population co-expressed the higher levels of CD11c and CD14, compared to FSC low cells, possibly indicative of their activation status. (B, C) Representative flow cytometry plots from each patient from the 4 groups of patients and one healthy control are presented according to gating strategies shown in A. (D) Comparative assessment of the frequency of each myeloid cell population among healthy controls (n=5), RRMS patients in remission (n=4), RRMS patients in relapse (n=4), TMS patients in remission (n=5) and TMS patients in relapse (n=5). RRMS, Relapsing Remitting Multiple Sclerosis; Rem, Remission; Rel, Relapse; TMS, Tumefactive Multiple Sclerosis; HD, Healthy donors; FSC, forward side scatter; PBMC, peripheral blood mononuclear cells. [file Image_11.tif]

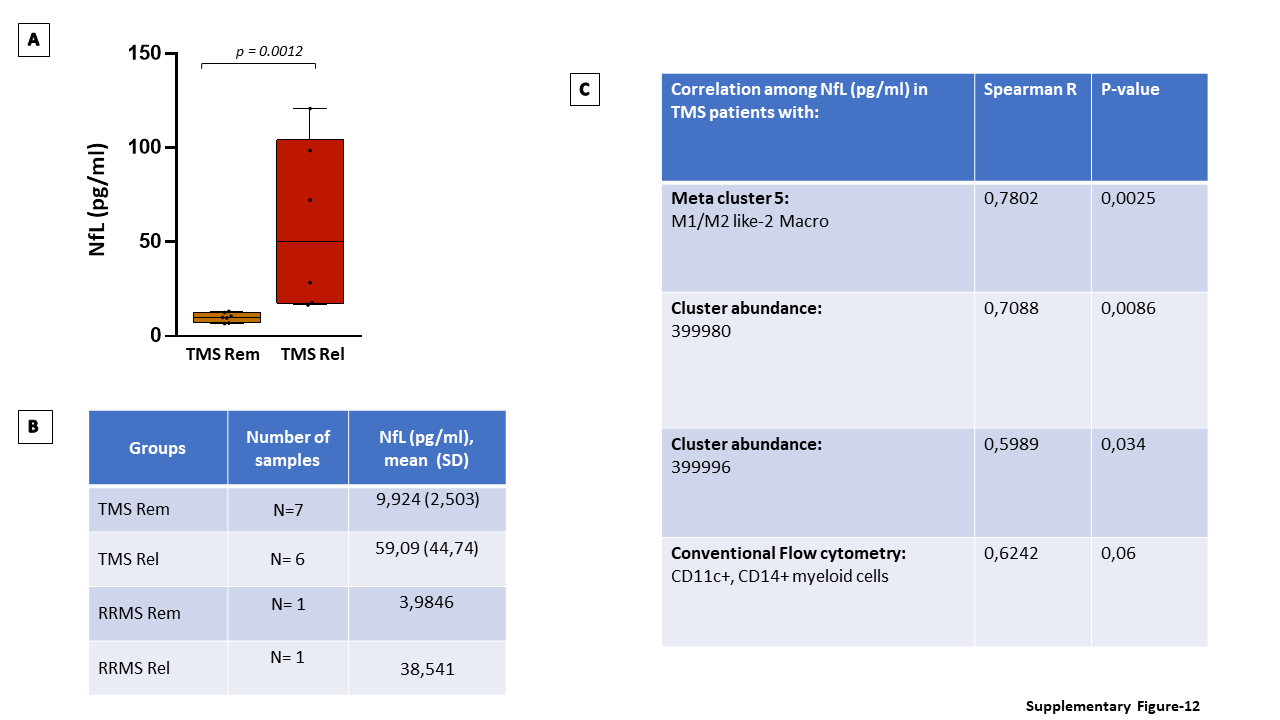

Supplement: Supplementary Figure 12 — Increased serum neurofilament light chains reflect disease activity in TMS patients and correlate with the expansion of the myeloid cell signature. (A) Serum neurofilament light chains were measured in duplicates using the high-sensitivity single-molecule array (Simoa) NF-Light Advantage Kit (Quanterix), according to the manufacturer’s instructions on an HDX platform (Quanterix). (B) Serum NfL in RRMS and TMS patients. (C) Correlation among NfL and myeloid cell signatures as defined by analysis of CytOF (also see ) and conventional flow cytometry data analysis. RRMS, Relapsing-Remitting Multiple Sclerosis; TMS, Tumefactive Multiple Sclerosis; Macro, macrophages; Rem, Remission; Rel, Relapse; NfL, neurofilament light chains; SD, standard deviation. [file Image_12.tif]

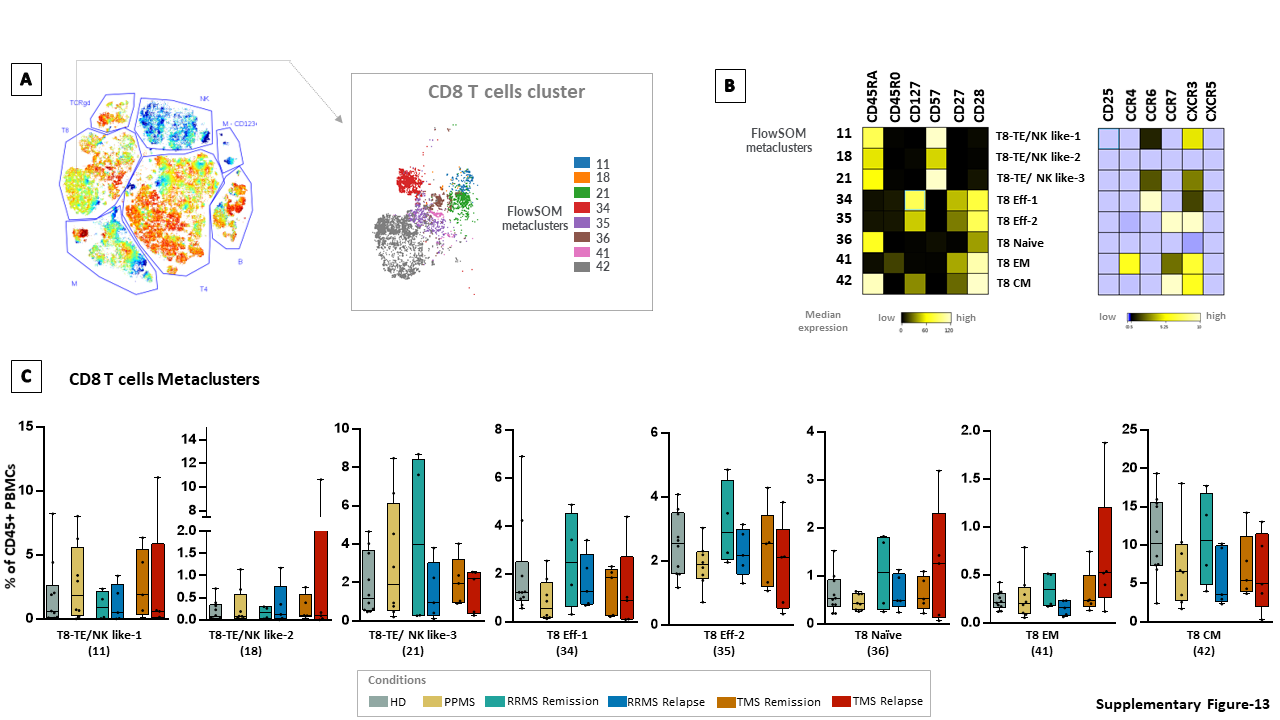

Supplement: Supplementary Figure 13 — Differences in CD8+ T cells among MS disease subgroups and healthy controls. (A) FlowSOM generated metaclusters for the CD8+ T cell lineage projected on the tSNE map. (B) Heatmap showing, for each of the clusters generated, the average intensity of each lineage marker across all cells. A tentative biological name was assigned to each metacluster based on the most abundant CD8+ T cell related marker expressed by each cluster. (C) MS patients stratified in different disease subgroups (PPMS; n=8, RRMS; n=9, TMS; n=10). RRMS and TMS were further subdivided in those in remission (RRMS; n=4, TMS; n=5) and relapse (RRMS; n=5, TMS; n=5). Box plots showing the frequency (expressed as % of CD45+ cells) of each major immune cell subset in peripheral blood mononuclear cells of patient subgroup and healthy donors (n=10). Each dot represents the value of each sample. p < 0.05 was considered significant, non-parametric Kruskal Wallis test with correction for multiple comparisons (FDR) was applied. PPMS, Primary Progressive Multiple Sclerosis; RRMS, Relapsing Remitting Multiple Sclerosis; Rem, Remission; Rel, Relapse; HD, Healthy donors; TMS, Tumefactive Multiple Sclerosis; FlowSOM, Flow Self-Organizing Map; FDR, false discovery rate. [file Image_13.tif]

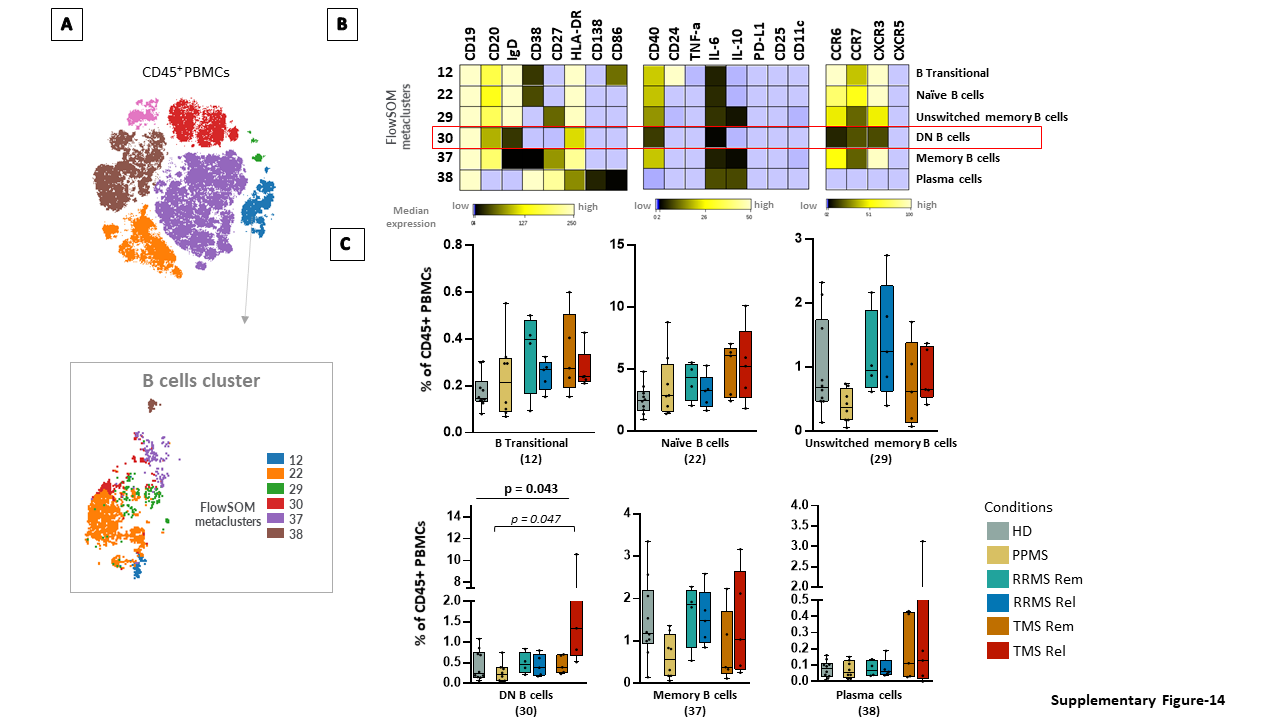

Supplement: Supplementary Figure 14 — Higher abundance of a memory double negative B cell subset in TMS patients during relapse. (A) FlowSOM generated metaclusters for the B cell lineage projected on the tSNE map. (B) Heatmap showing, for each of the metaclusters generated, the average intensity of each B cell related marker (stage or activation marker). A tentative biological name was assigned to each metacluster based on the most abundant B cell related marker expressed by each cluster. (C) MS patients stratified in different disease subgroups (PPMS; n=9, RRMS; n=9, TMS; n=10). RRMS and TMS were further subdivided in those in remission (RRMS; n=4, TMS; n=5) and relapse (RRMS; n=5, TMS; n=5). Box-plots show frequencies (%) of the indicative cell types. Each dot represents the value of each sample. p < 0.05 was considered significant, non-parametric Kruskal Wallis test with correction for multiple comparisons (FDR) was applied. PPMS, Primary Progressive Multiple Sclerosis; RRMS, Relapsing Remitting Multiple Sclerosis; Rem, Remission; Rel, Relapse; TMS, Tumefactive Multiple Sclerosis; HD, Healthy donors, t-SNE, t-distributed stochastic neighbor embedding; FlowSOM, Flow Self-Organizing Map; PD-L1, Programmed death-ligand 1; TNF-α, tumor necrosis factor alpha; IL-10, Interleukin 10; IL-6, Interleukin 6; CCR, CC chemokine receptors; CXCR, CXC chemokine receptor; FDR, false discovery rate. [file Image_14.tif]

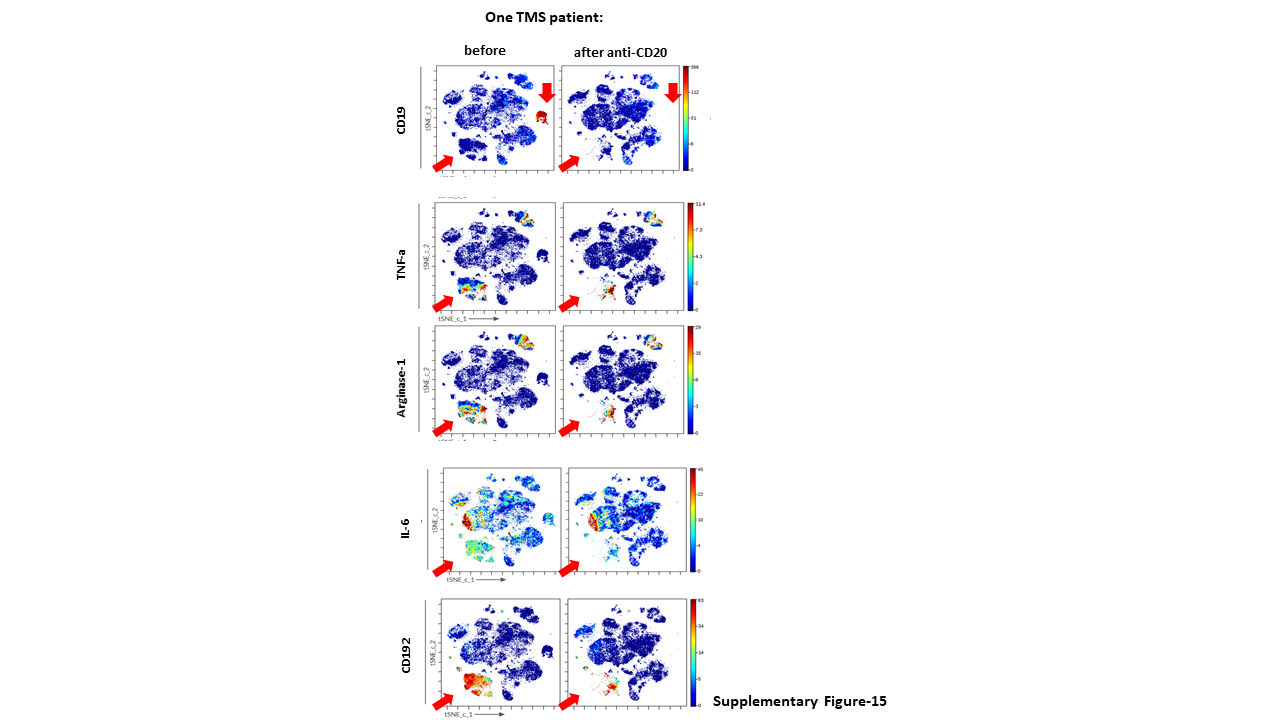

Supplement: Supplementary Figure 15 — tSNE plots of one TMS patient during relapse and after anti-CD20 treatment. TMS, Tumefactive Multiple Sclerosis; CCR2 or CD192, C-C chemokine receptor type 2; TNF-α, tumor necrosis factor alpha; IL-6, Interleukin 6. [file Image_15.tif]

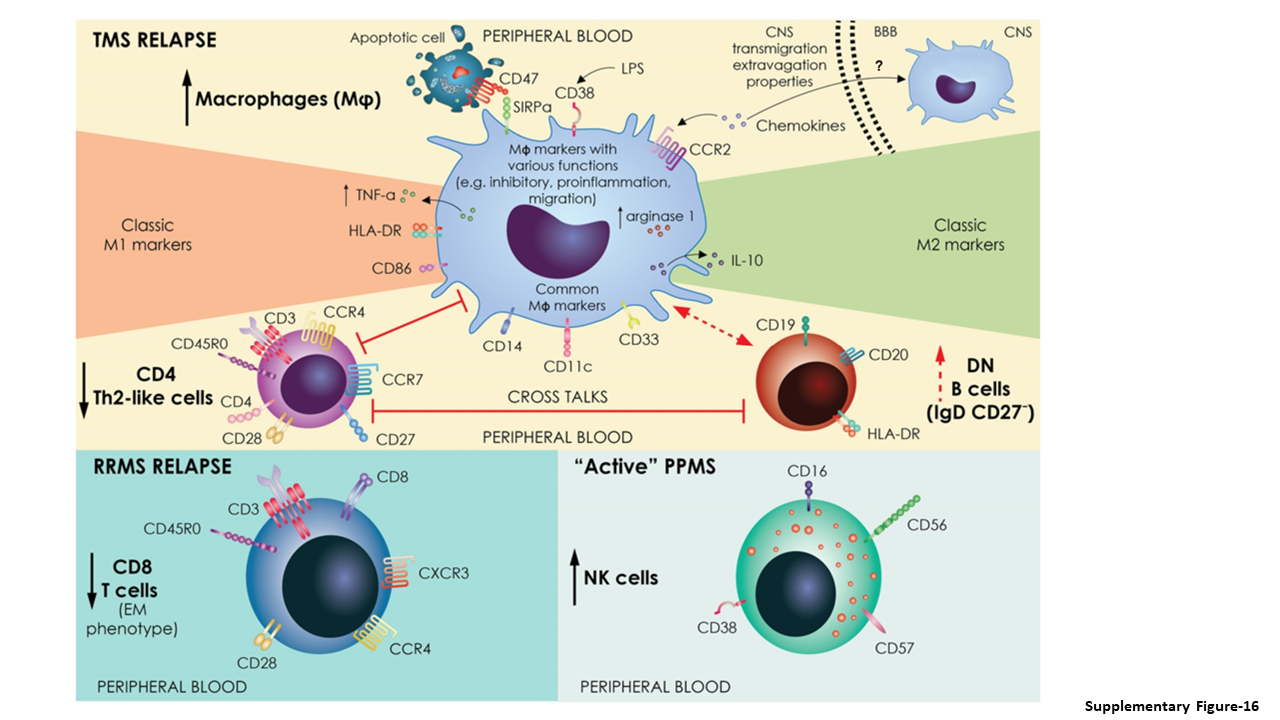

Supplement: Supplementary Figure 16 — Schematic representation of cell-types differentially expressed in peripheral blood as revealed by broad immunophenotyping with CyTOF analysis. The cross-talks among cell types are based on statistically significant correlations as revealed by correlation matrix among all of immune subsets in relapsing MS (RRMS and TMS) patients as shown in . The image is hypothesis driven and implicates different cell drivers in different clinical subtypes of MS patients. PPMS, Primary Progressive Multiple Sclerosis; RRMS, Relapsing Remitting Multiple Sclerosis; TMS, Tumefactive Multiple Sclerosis. [file Image_16.tif]
