# Supplementary material for: Specific myeloid signatures in peripheral blood differentiate active and rare clinical phenotypes of multiple sclerosis
Source: Front Immunol. 2023 Jan 25;14:1071623. doi: 10.3389/fimmu.2023.1071623 (PMC9905713; doi:10.3389/fimmu.2023.1071623)
Supplement: Supplementary file 17 [file Table_1.docx]

**Supplementary Table 1.** **Antibodies used for mass cytometry and conventional flow cytometry staining**

|  | **Target** | **Clone** | | **Metal or fluorochromes** | **Source** | **Localization** | **Dilution** |
| --- | --- | --- | --- | --- | --- | --- | --- |
| 1 | **CD45** | HI30 | | 89 Y | MDIPA backbone SB | Cell-surface | N/A |
| 2 | **CCR6** | G034E3 | | 141 Pr | MDIPA backbone SB | Cell-surface | N/A |
| 3 | **CD123** | 6H6 | | 143 Nd | MDIPA backbone SB | Cell-surface | N/A |
| 4 | **CD19** | HIB19 | | 144 Nd | MDIPA backbone SB | Cell-surface | N/A |
| 5 | **CD4** | RPA‐T4 | | 145 Nd | MDIPA backbone SB | Cell-surface | N/A |
| 6 | **CD8a** | RPA‐T8 | | 146 Nd | MDIPA backbone SB | Cell-surface | N/A |
| 7 | **CD11c** | Bu15 | | 147 Sm | MDIPA backbone SB | Cell-surface | N/A |
| 8 | **CD16** | 3G8 | | 148 Nd | MDIPA backbone SB | Cell-surface | N/A |
| 9 | **CD45RO** | UCHL1 | | 149 Sm | MDIPA backbone SB | Cell-surface | N/A |
| 10 | **CD45RA** | HI100 | | 150 Nd | MDIPA backbone SB | Cell-surface | N/A |
| 11 | **CD161** | HP‐3G10 | | 151 Eu | MDIPA backbone SB | Cell-surface | N/A |
| 12 | **CCR4** | L291H4 | | 152 Sm | MDIPA backbone SB | Cell-surface | N/A |
| 13 | **CD25** | BC96 | | 153 Eu | MDIPA backbone SB | Cell-surface | N/A |
| 14 | **CD27** | O323 | | 154 Sm | MDIPA backbone SB | Cell-surface | N/A |
| 15 | **CD57** | HCD57 | | 155 Gd | MDIPA backbone SB | Cell-surface | N/A |
| 16 | **CXCR3** | G025H7 | | 156 Gd | MDIPA backbone SB | Cell-surface | N/A |
| 17 | **CXCR5** | J252D4 | | 158 Gd | MDIPA backbone SB | Cell-surface | N/A |
| 18 | **CD28** | CD28.2 | | 160 Gd | MDIPA backbone SB | Cell-surface | N/A |
| 19 | **CD38** | HB‐7 | | 161 Dy | MDIPA backbone SB | Cell-surface | N/A |
| 20 | **CD56** | NCAM16.2 | | 163 Dy | MDIPA backbone SB | Cell-surface | N/A |
| 21 | **TCRgd** | B1 | | 164 Dy | MDIPA backbone SB | Cell-surface | N/A |
| 22 | **CD294** | BM16 | | 166 Er | MDIPA backbone SB | Cell-surface | N/A |
| 23 | **CCR7** | G043H7 | | 167 Er | MDIPA backbone SB | Cell-surface | N/A |
| 24 | **CD14** | 63D3 | | 168 Er | MDIPA backbone SB | Cell-surface | N/A |
| 25 | **CD3** | UCHT1 | | 170 Er | MDIPA backbone SB | Cell-surface | N/A |
| 26 | **CD20** | 2H7 | | 171 Yb | MDIPA backbone SB | Cell-surface | N/A |
| 27 | **CD66b** | G10F5 | | 172 Yb | MDIPA backbone SB | Cell-surface | N/A |
| 28 | **HLADR** | | LN3 | 173 Yb | MDIPA backbone SB | Cell-surface | N/A |
| 29 | **IgD** | | IA6‐2 | 174 Yb | MDIPA backbone SB | Cell-surface | N/A |
| 30 | **CD127** | | A019D5 | 176 Yb | MDIPA backbone SB | Cell-surface | N/A |
| 31 | **CD192** | | K036C2 | 106 Cd | In-house | Cell-surface | 1/300  (stock: 0.5 mg/ml) |
| 32 | **CD206** | | 15-2 | 110 Cd | In-house | Cell-surface | 1/150  (stock: 0.5 mg/ml) |
| 33 | **CD172a/b** | | SE5A5 | 111 Cd | In-house | Cell-surface | 0.4/300  (stock: 0.5 mg/ml) |
| 34 | **TNFa** | | MAb11 | 112 Cd | In-house | Intracellular | 1/300  (stock: 0.5 mg/ml) |
| 35 | **CD24** | | ML5 | 113 Cd | In-house | Cell-surface | 1/150  (stock: 0.5 mg/ml) |
| 36 | **Arginase-1** | | 14D2C43 | 116 Cd | In-house | Intracellular | 1/300  (stock: 0.5 mg/ml) |
| 37 | **CD138** | | DL-101 | 163 Dy | In-house | Cell-surface | 1/150  (stock: 0.5 mg/ml) |
| 38 | **CD86** | | IT2.2 | 114 Cd | In-house | Cell-surface | 1/150  (stock: 0.5 mg/ml) |
| 39 | **IL-10** | | JES3- 9D7 | 159 Tb | In-house | Intracellular | 1/150  (stock: 0.5 mg/ml) |
| 40 | **IL-6** | | MQ2- 13A5 | 175 Lu | In-house | Intracellular | 1/150  (stock: 0.5 mg/ml) |
| 41 | **CD40** | | 5C3 | 142 Nd | In-house | Cell-surface | 1/300  (stock: 0.5 mg/ml) |
| 42 | **CD33** | | WM53 | 169 Tm | In-house | Cell-surface | 0.4/300  (stock: 0.5 mg/ml) |
| 43 | **CD274/**  **PD-L1** | | MIH1 | 209 Bi | In-house | Cell-surface | 1/300  (stock: 0.5 mg/ml) |
| 44 | **CD163** | | GHI/61 | 165 Ηο | In-house | Cell-surface | 1/300  (stock: 0.5 mg/ml) |
| Flow cytometry | | | | | | | |
| 1 | **CD11c** | | Bu15 | APC | Biolegend | Cell-surface | 1/300 |
| 2 | **CD14** | | 63D3 | FITC | Biolegend | Cell-surface | 1/300 |
| 3 | **CD3/19/56** | | OKT4/ HIB19/ QA17A16 | PE-Cy7 | Biolegend | Cell-surface | 1/300 |

Abbreviations: N/A; non-applicable, SB; Standard Biotools Inc. (formerly Fluidigm)
