# Supplementary material for: Specific myeloid signatures in peripheral blood differentiate active and rare clinical phenotypes of multiple sclerosis
Source: Front Immunol. 2023 Jan 25;14:1071623. doi: 10.3389/fimmu.2023.1071623 (PMC9905713; doi:10.3389/fimmu.2023.1071623)
Supplement: Supplementary file 18 [file Table_2.docx]

Supplementary Table 2

MRI features of PPMS patients with emphasis on the presence of leptomeningeal enhancement.

|  | | | **Conformation of LMCE** | |  |
| --- | --- | --- | --- | --- | --- |
| **A/A** | **PPMS patients** | **Number of foci (LMCE)** | **Nodular** | **Flat / Linear** | **Brain region of foci** |
| **1** | PPMS-1 | 0 | N/A | N/A | N/A |
| **2** | PPMS-2 | 1 | 1 | 0 | occipital |
| **3** | PPMS-3 | 0 | N/A | N/A | N/A |
| **4** | PPMS-4 | 0 | N/A | N/A | N/A |
| **5** | PPMS-5 | 0 | N/A | N/A | N/A |
| **6** | PPMS-6 | 1 | 1 | 0 | frontal |
| **7** | PPMS-7 | 1 | 1 | 0 | 1 parietoccipital |
| **8** | PPMS-8 | 2 | 2 | 0 | 1 temporal, 1 occipital |

Abbreviations: PPMS: Primary progressive multiple sclerosis; MRI: magnetic resonance imaging, LMCE: leptomeningeal contrast enhancement, 3D-FLAIRGd: three-dimensional fluid-attenuated inversion recovery post-gadolinium sequence, N/A: non-applicable
