# Supplementary material for: Specific myeloid signatures in peripheral blood differentiate active and rare clinical phenotypes of multiple sclerosis
Source: Front Immunol. 2023 Jan 25;14:1071623. doi: 10.3389/fimmu.2023.1071623 (PMC9905713; doi:10.3389/fimmu.2023.1071623)
Supplement: Supplementary file 19 [file Table_3.docx]

|  | **Follow-up data** | **Available Biopsy** | **Diagnosis on Biopsy** |
| --- | --- | --- | --- |
| Pt1 | A new relapse occurred, in remission under anti-CD20 treatment | Yes | Inflammatory demyelinating disease,  compatible with MS |
| Pt2 | A new relapse occurred, in remission under anti-CD20 treatment | No | N/A |
| Pt3 | In remission under anti-CD20 treatment, with residual neurological deficits | No | N/A |
| Pt4 | Stable in remission under anti-CD20 (partial deterioration upon anti-CD20 initiation) | No | N/A |
| Pt5 | Started anti-CD20 and is stable in remission | Yes | Inflammatory demyelinating disease, compatible with MS |
| Pt6 | Free of relapse, stable | No | N/A |
| Pt7 | Stable and in remission under anti-CD20 treatment | No | N/A |
| Pt8 | Lost in follow-up | Yes | Inflammatory demyelinating disease, compatible with MS |
| Pt9 | Started anti-CD20, after 2 cycles in remission | No | N/A |
| Pt10 | Stable and in remission under anti-CD20 (partial deterioration upon anti-CD20 initiation) | No | N/A |
| Pt11 | Stable, started Natalizumab | No | N/A |
| Pt12 | Started anti-CD20 and after this in remission | Yes | Inflammatory demyelinating disease, compatible with MS |
| Pt13 | Started anti-CD20 therapy and after this in remission | No | N/A |
| Pt14 | Started anti-CD20 and after in remission with residual neurological deficits | No | N/A |

Abbreviations: N/A; non-applicable, Pt; patient, anti-CD20; Anti-CD20 monoclonal antibodies for B cell depletion, MS; Multiple Sclerosis
